# Dual biocontrol and osmotic stress mitigation by endophytic *Aspergillus micronesiensis* and *Penicillium momoi* against fusarium pathogens

**DOI:** 10.1371/journal.pone.0353217

**Published:** 2026-07-29

**Authors:** Ali Aligholizadeh, Mina Salehi, Seyed Bagher Mahmoudi, Naser Safaie

**Affiliations:** 1 Department of Plant Pathology, Faculty of Agriculture, Tarbiat Modares University, Tehran, Iran; 2 Department of Plant Genetics and Breeding, Faculty of Agriculture, Tarbiat Modares University, Tehran, Iran; 3 Sugar Beet Seed Institute, Agricultural Research, Education, and Extension Organization (AREEO), Karaj, Iran; University of Duhok, IRAQ

## Abstract

Global challenges associated with crop diseases and abiotic stress necessitate sustainable agricultural solutions. This study investigated the biocontrol and osmotic-stress mitigation potential of two endophytic fungi, which were isolated from native plants and subsequently identified as *Aspergillus micronesiensis* and *Penicillium momoi*. Their antagonistic activities against *Fusarium oxysporum* f. sp. *lycopersici* (FOL), *F. oxysporum* f. sp. *radicis-lycopersici* (FORL), and *F. pseudograminearum* (FPS) were evaluated. Complementary antagonistic mechanisms were observed: *P. momoi* primarily inhibited pathogen growth through direct mycelial competition, while *A. micronesiensis* predominantly exerted effects through antibiosis. In dual-culture assays, *A. micronesiensis* inhibited FOL, FORL, and FPS by 39.05%, 39.93%, and 54.59%, respectively, whereas *P. momoi* caused inhibition rates of 51.84%, 64.74%, and 60.66%, respectively, demonstrating strong biocontrol potential. The salt tolerance assay revealed that both endophytes were able to grow on media containing up to 3 M NaCl. Although, optimal growth for both isolates occurred at 1 M NaCl, *A. micronesiensis* showed a greater increase in growth relative to the control (2.06 times). In contrast, *P. momoi* maintained more consistent growth across the 0–1 M NaCl range. However, growth of both isolates declined at higher salt concentrations. The developed wettable powder formulation maintained high spore viability for at least 27 months under room-temperature conditions. This research highlights *A. micronesiensis* and *P. momoi* as promising agents for managing Fusarium diseases and enhancing plant salinity tolerance. Further metabolomics investigations are recommended to clarify the functional roles of these endophytes in crop protection under stress conditions.

## 1. Introduction

Endophytic fungi are microorganisms colonizing internal plant tissues without causing disease symptoms, providing multiple benefits to their host plants through enhanced plant growth, improved nutrient acquisition, and increased tolerance to both biotic and abiotic stresses [1–5]. These symbionts have attracted attention in sustainable agriculture, particularly through seed-coating technologies that facilitate early microbial colonization and improve seedling establishment and vigor [6,7]. Such approaches reduce reliance on chemical inputs while promoting natural plant-microbe interactions and supporting sustainable agricultural practices [8,9].

One of the principal abiotic stresses limiting crop productivity worldwide is soil salinity. Saline soils reduce water availability and disrupt nutrient balance, adversely affecting plant growth and yield [10–12]. Endophytic fungi have demonstrated remarkable tolerance to salinity and can alleviate salt stress through physiological mechanisms such as osmolyte accumulation and antioxidant production [5,13,14]. Additionally, these fungi improve nutrient uptake and produce phytohormones that contribute to plant resilience under saline conditions [15,16].

Tomato Fusarium wilt, caused by *Fusarium oxysporum* f. sp. *Lycopersici* [17,18], and wheat crown rot, caused primarily by *F. pseudograminearum* and *F. culmorum* [19–21], are significant soil-borne diseases that threaten crop productivity globally. These pathogens not only reduce yield but also produce mycotoxins that pose a threat to food safety [22–24]. Therefore, integrated disease management strategies increasingly emphasize biological control approaches for sustainable crop protection. Biological control using microbial biological control agents (MBCAs) can suppress pathogens, reduce disease severity, and enhance plant tolerance to abiotic stresses, such as salinity [5]. The use of these beneficial fungi supports sustainable agriculture by reducing reliance on chemical fungicides and improving soil health.

*Penicillium* and *Aspergillus* are important MBCAs utilized in agriculture to suppress plant pathogens such as *Fusarium* [25]. These fungi produce antifungal metabolites that inhibit pathogen growth and reduce disease severity. Additionally, they enhance plant tolerance to abiotic stresses, such as salinity, by improving nutrient uptake and stimulating plant defense mechanisms [26,27].

Endophyte-based seed coatings have emerged as promising biocontrol tools by delivering beneficial microbes directly to seeds. This technology promotes early colonization by endophytic fungi that not only antagonize pathogen through multiple biocontrol mechanisms, but also enhance plant tolerance to abiotic stresses such as salinity [9,28–30]. Optimizing seed coating formulations that consider factors such as microbial viability, adhesive properties, and controlled release is crucial for ensuring prolonged, adequate protection during plant growth [31].

This study aimed to taxonomically identify the causal agents of Tomato Fusarium wilt and wheat crown rot, as well as fungal biocontrol agents “*Penicillium* sp. and *Aspergillus* sp.”, (2) evaluate their biocontrol potential against *Fusarium oxysporum* f. sp. *lycopersici*, *F. o.* f. sp. *radicis-lycopersici*, and *F. pseudograminearum*, and (3) develop optimized formulations ensuring long-term viability to support practical agricultural applications. This integrated approach combines antagonistic mechanisms and osmotic resilience to support sustainable crop management under stress conditions.

## 2. Materials and Methods

### 2.1. Isolation and identification of pathogenic fungi

Infected plant samples were collected in 2019−2024 from wheat fields in Shahin Dezh (West Azerbaijan), showing crown and root rot, and from tomato plants in East Azerbaijan with Fusarium wilt symptoms. The method of Ma et al. [32] was followed with slight modifications for the isolation of pathogenic fungi. First, the crown and root portions of the samples were washed under running tap water to remove excess soil. Tissues (3–5 mm) from the infected crown, stem, and root regions were surface-sterilized with 70% ethanol (v/v) for 2 min, followed by three rinses with sterile distilled water (2 min each), and subsequently immersed in 96% ethanol for 40 s. The surface-disinfected pieces were placed on potato dextrose agar (PDA) supplemented with 250 mg l^-1^ Chloramphenicol and incubated at 27 ± 2 °C. Emerging hyphae were subcultured on PDA and purified using the hyphal-tip method on water agar (WA). Pathogenicity tests were performed on sterilized seeds of four wheat cultivars (Sardari, Homa, Sadra, and Hashtrood) and on tomato seedlings of cultivar Super chief in sterile soil inoculated with fungal material. Disease severity was assessed one month after inoculation by evaluating symptom development and growth reduction. The most virulent wheat isolates were identified by PCR amplification of the ITS region [33] and β-tubulin [34; S1 Table]. At the same time, universal primers (UniF and UniR [35]) and specific primers (sprlF and sprlR [35]) were used for identifying the tomato isolates (S1 Table). Genomic DNA was extracted from frozen mycelia (40–50 mg) as described previously [36]. The thermal program included initial denaturation at 94 °C for 5 min; 35 cycles of denaturation at 94 °C for 1 min, annealing at 55 °C for ITS, 58 °C for β-tubulin, and 62 °C for tomato-specific primers for 1 min, and extension at 72 °C for 2 min; followed by a final extension at 72 °C for 5 min. PCR products were separated using agarose gel electrophoresis and subsequently purified with a DNA gel extraction kit (Axygen Biotechnology Ltd., China). These purified fragments were then sequenced directly by Bioneer (Shanghai, China) using the same primers applied in the PCR reactions.

### 2.2. Morphological and Molecular identification of biocontrol fungi

*Penicillium* sp. (isolated from *Astragalus squarrosus* leaf) and *Aspergillus* sp. (isolated from *Gypsophila mucronifolia* leaf) were obtained from the Laboratory of Plant Diseases Bicontol and Management, Department of Plant Pathology, Faculty of Agriculture, Tarbiat Modares University, Tehran, Iran.

For morphological characterization, both fungal isolates were cultured on six different agar media: Czapek Yeast Autolysate agar (CYA), Czapek’s agar (CZA), Malt Extract agar (MEA), Oatmeal agar (OA), Yeast Extract Sucrose agar (YESA), and PDA (S2 Table; [37]). Cultures were incubated at 27 ± 2 °C in the dark for 14 days in triplicate. Colony morphology, growth rate, and microscopic features (e.g., conidia, metulae, phialides, conidiophores, vesicles, and stipes) were analyzed. Microscopic observations were performed using Lactophenol-cotton blue-stained slides, and images were captured with an Olympus BX51 microscope equipped with a DP72 camera.

The PCR reaction mixtures (25 μl) consisted of 12.5 μl Premix Taq (TaKaRa Biotechnology Ltd., Japan), one μl of genomic DNA (~100 ng), one μl of forward and reverse primers (10 Pmol), and 10.5 μl PCR-grade water. The PCR reaction programs were an initial denaturation at 94 °C for 3 min, followed by 30 cycles of denaturation (94 °C for 30 s), annealing (56 °C (ITS) [33], and 55 °C (TUB [34], RPB2 [38], and CMD [39]) for 30 s), extension (72 °C for 1 min) and a final extension at 72 °C for 5 min. The PCR products were confirmed by agarose gel electrophoresis and then purified using a DNA gel extraction kit (Axygen Biotechnology Ltd., China). The purified PCR product was directly sequenced using the same primers by Bioneer (Shanghai, China).

The alignments of combined ITS and TUB/ITS, TUB, CMD, and RPB2 sequences of the strains in this study and those obtained from GenBank (S3 and S4 Tables) were compared. Sequences were aligned using MUSCLE in MEGA v11 and manually refined in the MEGA software by deleting the headers and footers until the maximum number of shared sequences was reached. *Hamigera avellanea* and *H. brevicompacta* were added as outgroup taxa for *Aspergillus* sp. and *Penicillium* sp. [40]. Also, *Fusarium nelsonii* was designated as the outgroup taxon for *F. pseudograminearu*m [41]. Analysis was performed based on Bayesian inference (BI). The phylogenetic analysis of *Aspergillus* sp. and *Penicillium* sp. was performed using the GTR + G + I model, which accounts for invariant sites. On the other hand, the phylogenetic tree for *F. pseudograminearu*m was constructed using the SYM + G model. The Bayesian analyses were performed using MrBayes v3.1.2 [42]. with a random starting tree, and the chains were run for 5 million generations. After discarding burn-in samples and evaluating convergence, the remaining samples were retained for further analyses. The Markov chain Monte Carlo (MCMC) method, within a Bayesian framework, was used to estimate the posterior probabilities of the phylogenetic trees [43] using the 50% majority rule. The convergence of model parameters and topology was assessed based on the average standard deviation of split frequencies and the potential scale reduction factor. A maximum likelihood (ML) tree was reconstructed with RaxmlGUI 1.1 [44] using the same nucleotide substitution model for BI, and 1000 bootstrap (BS) pseudo-replicates were generated. The output file of the phylogenetic program was visualized using Dendroscope V.3.2.8 [45] and annotated in PowerPoint.

### 2.3. Salt tolerance assay

The fungal growth was assessed on PDA supplemented with different NaCl concentrations (0, 1, 2, and 3 M). Cultures were incubated at 27 ± 2 °C in darkness [46] and radial growth was measured after 14 days. The experiment was designed as a factorial based on a randomized complete block design (RCBD) with five replicates.

### 2.4. Antagonistic activity assays

Antagonistic activity against pathogenic fungi was assessed using a dual culture assay. Biocontrol isolates of *Penicillium* sp. and *Aspergillus* sp. were first cultured on PDA plates for 7 days. Subsequently, mycelial discs (5 mm diameter) of the pathogens *F. oxysporum* f. sp. *lycopersici*, *F. radicis-lycopersici*, and *F. pseudograminearum* were placed on the opposite side of the same plate. Control plates contained only fungal pathogen discs. All plates were incubated at 27 ± 2 °C in the dark.

The percentage inhibition of radial growth (PIRG) was estimated using Eq. (1).


$$\begin{array}{c} PIRG=\left(\frac{R_{\text{1}}-R_{\text{2}}}{R_{\text{1}}}\right)\;\times \text{1}\text{0}\text{0} \end{array}$$ (1)

where R_1_ denotes the pathogen colony radius in the control plate, and R_2_ is the radius of the pathogen colony in dual culture.

The experiment was designed as a factorial RCBD with four replicates.

### 2.5. Formulation preparation

Wheat grains were hydrated either by boiling for 20–30 min, followed by autoclaving twice at 121 ºC for 40 min. The sterilized grains were inoculated with 2 agar discs (5 mm diameter) containing mycelia of biocontrol isolates, grown on PDA, and incubated at 27 ± 2 °C for 15 days with regular agitation to prevent clumping. Spores were harvested in sterile water, and the concentration was adjusted to 6 × 10^5^ (for *Aspergillus* sp.) and 3 × 10⁹ (for *Penicillium* sp.) spores ml^-1^ using a hemocytometer.

Formulations were prepared by thoroughly mixing spore suspensions (48% v/v) with selected carriers (45% w/v; wheat bran, sodium alginate, sodium bentonite, and talc), stabilizers (0.04% w/v; sodium nitrate and dipotassium hydrogen phosphate), Tween 20 (3% v/v) as a wetting agent, and carboxymethyl cellulose (CMC) as a suspending agent. Detailed compositions of all formulations are presented in S5 Table.

The mixture was dried at 38.5 °C using a germinator, then ground into powder and stored in sealed zip-lock bags.

### 2.6. Optimal carrier selection and stability assessment

Eighteen different formulations (S5 Table) were evaluated by varying carriers and stabilizers while keeping Tween 20 and CMC constant.

Formulation stability was assessed by serial dilution plating (up to an eightfold dilution) at intervals ranging from 1 to 27 months post-production. Spore viability was determined by counting germinated colonies on PDA plates. Colony-forming units (CFU) were quantified via serial dilution plating on PDA. Two independent experiments were set up in an RCBD with three replicates.

### 2.7. Greenhouse evaluation of optimized formulation of biocontrol agents under combined drought and pathogen stress

To evaluate the performance of optimized formulation of biocontrol agents “*Penicillium* sp. and *Aspergillus* sp. under greenhouse conditions, a factorial experiment was conducted using a RCBD with four replications. The study assessed the interactive effects of wheat cultivar, drought stress, pathogen inoculation, and treatment type on plant growth and disease development.

The experimental factors included: (A) wheat cultivar (Hashtrood and Sadra), (B) drought stress (well-watered control and drought stress), (C) pathogen inoculation (non-inoculated and inoculated with *F. pseudograminearum*), and (D) treatment type with five levels: optimized formulation based on *Aspergillus* sp. (AspF), optimized formulation based on *Penicillium* sp. (PenF), empty formulation without biological agent (EmpF), EmpF combined with tebuconazole (EmpF + TEB), and tebuconazole alone (TEB). This structure allowed separation of biological agent effects, empty formulation effects, and chemical fungicide efficacy.

Field soil was sieved and sterilized twice at 121 °C for 24 h before being air-dried. Inoculum of *F. pseudograminearum* was prepared using autoclaved wheat seeds colonized with 5-mm mycelial plugs from 7-day-old PDA culture incubated at 27 ± 2 °C. After full colonization, excess moisture was removed aseptically. Colonized seeds were mixed with the upper soil layer at 1% (w/w) and transferred into 2-L pots. Wheat seeds of Hashtrood and Sadra cultivars were surface-sterilized with 0.6% sodium hypochlorite for 2 min, followed by 70% ethanol for 2 min, and rinsed three times with sterile distilled water. Seeds were then coated with the respective treatments prior to sowing. Two seeds were sown per pot and maintained under greenhouse conditions with uniform irrigation.

The soil moisture content was maintained within the ranges of 35–45% and 15–25% for the well-watered control and drought-stress treatments, respectively, using a modified method described by Imakumbili [47].

Disease score was recorded 45 days after inoculation using a 0–4 scaleas described previously [48]. Growth parameters, including plant height (measured from the soil surface to the tip of the main stem in cm), SPAD chlorophyll index (measured using a SPAD meter on the uppermost fully expanded leaf), and shoot and root fresh and dry weights (measured in grams after drying at 70 °C to constant weight), were also determined.

The experiments were conducted in the research greenhouses of the Department of Plant Pathology at Tarbiat Modares University (TMU), and no permission was required.

### 2.8. Statistical analysis

Normality of data and homogeneity of variances were verified. Following confirmation of data normality and homogeneity of variances (especially after data transformation for disease score), parametric tests were applied. Disease scores were initially converted to proportions by dividing the 0–4 scale by 4, yielding values ranging from 0 to 1. Subsequently, these proportions were transformed using the arcsin-square root transformation (arcsin(√(p))), where p represented the proportion of disease score. Analysis of variance (ANOVA) and mean comparisons using the least significant difference (LSD) test were performed using SAS and SPSS. GraphPad software was used to generate graphical representations of the data.

## 3. Results

### 3.1. Molecular identification of *Fusarium* pathogens

Using the specific primers (Uni and Spr1), two isolates of *F. oxysporum*, namely *F. o.* f. sp. *lycopersici* and *F. o.* f. sp. *radicis-lycopersici*, were distinguished from other specific *F. oxysporum* species (S1–S3 Figs). Amplification with the Uni primer produced DNA fragments of 670–672 bp for both isolates (S2 Fig). One of the isolates did not produce any amplicon when tested with the Sprl primer targeting *F. o.* f. sp. *lycopersici* races 1–3 (S3 Fig). However, one isolate obtained a 700 bp amplicon using the Spr1 primer, which is specific to *F. o.* f. sp. *radicis-lycopersici* (S3 Fig).

**Fig 1:**
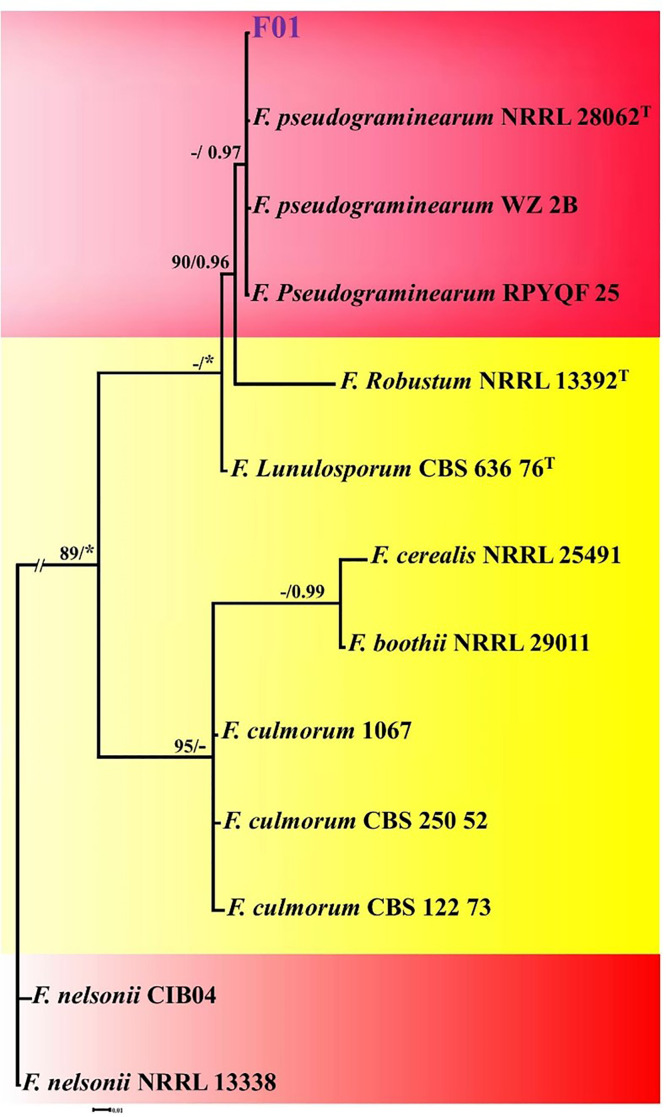
The phylogenetic tree, constructed using Bayesian 50% majority-rule consensus, employed the *ITS* and *TUB* gene regions from 50 nucleotide sequences under the SYM + G model. The most recently identified species, F01. *Fusarium nelsonii* (CIB04) and *F. nelsonii* (NRRL 13338), were designated as outgroup taxa. Bayesian posterior probabilities (BPP) exceeding 50% are provided for relevant clades. Ex-type strains are denoted by a superscript T. Bootstrap percentages from the maximum likelihood (ML) analysis and Bayesian inference (BI) posterior probability (pp) values are illustrated at the nodes (ML/pp). Values less than 70% bootstrap support (ML) or less than 0.95 posterior probability (Bayesian analysis) are indicated with a hyphen or not shown. Asterisks indicate full support (100% bootstrap or 1.00 pp). The bar indicates the frequency of substitutions per site.

Molecular characterization of the wheat crown and root rot causal agent used two genetic loci: *ITS* and *TUB*. Phylogenetic assessment using Bayesian and maximum-likelihood methods placed this isolate within the *F. pseudograminearum* clade, exhibiting considerable sequence homology across all genetic markers (S3 Table and Fig 1).

### 3.2. Morphological and microscopic identification of *Aspergillus* sp. and *Penicillium* sp

**Fig 2:**
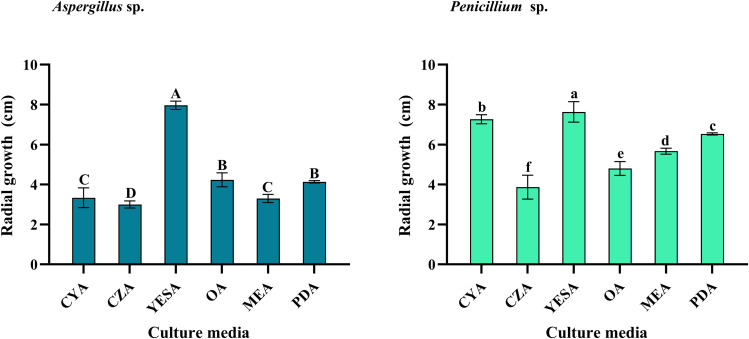
Radial growth of *Aspergillus* sp. and *Penicillium* sp. in Czapek yeast autolysate agar (CYA), Czapek’s agar (CZA), yeast extract sucrose agar (YESA), oatmeal agar (OA), malt extract agar (MEA), and potato dextrose agar (PDA) media. Values represent the mean ± standard error of *n =* 3 independent biological replicates. Significant differences are shown by different letters above the bars (*P <* 0.05).

**Fig 3:**
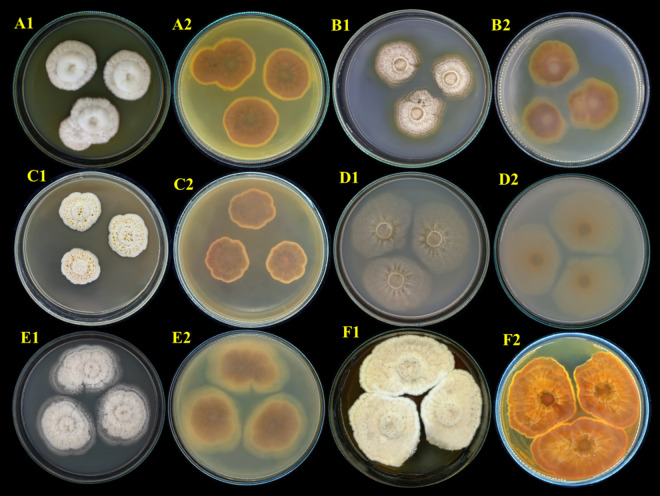
*Aspergillus* sp. on media cultures of Czapek Yeast Autolysate agar (CYA; A1 and A2), Czapek’s agar (CZA; B1 and B2), malt extract agar (MEA; C1 and C2), oatmeal agar (OA; D1 and D2), potato dextrose agar (PDA; E1 and E2), and yeast extract sucrose agar (YESA; F1 and F2). Indices 1 and 2 correspond to the top and bottom views, respectively, of the cultures on the 14^th^ day.

The radial growth of the endophytic *Aspergillus* sp. and *Penicillium* sp. was measured on six culture media—CYA, CZA, YESA, OA, MEA, and PDA—over 14 days at 27 ± 2 °C. Growth rates of *Aspergillus* sp. on these culture media varied significantly, with YESA showing the largest average colony diameter (7.97 cm), followed by OA (4.23 cm) and PDA (4.13 cm, Fig 2). CYA (3.33 cm) and MEA (3.30 cm) supported moderate growth (Fig 2). In comparison, CZA produced the smallest colonies (3.00 cm, Fig 2). Radial growth of *Aspergillus* sp. followed this order: YESA > OA = PDA > CYA = MEA > CZA. *Aspergillus* sp. colony morphology differed clearly among media: YESA colonies appeared dense, velvety, and intensely orange-to-reddish brown, with deep orange-brown reverse pigmentation (Fig 3), indicating optimal nutrient use. OA colonies were sparse and floccose with faint yellow pigmentation (Fig 3), suggesting limited growth. MEA and PDA supported moderate, velvety to powdery colonies with reddish-brown hues (Fig 3). Colonies on CYA and CZA were powdery-floccose or velvety-granular with pale colors (Fig 3).

**Fig 4:**
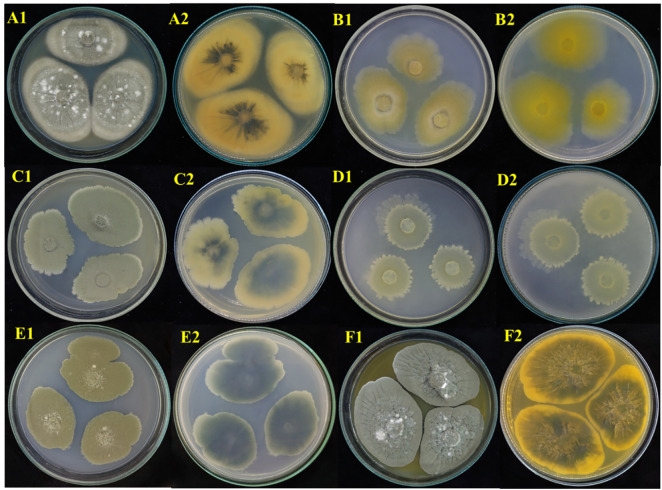
*Penicillium sp.* on media cultures of Czapek Yeast Autolysate agar (CYA; A1, A2), Czapek’s agar (CZA; B1, B2), malt extract agar (MEA; C1, C2), oatmeal agar (OA; D1, D2), potato dextrose agar (PDA; E1, E2), and yeast extract sucrose agar (YESA; F1 and F2). Indices 1 and 2 correspond to the top and bottom views, respectively, of the cultures on the 14^th^ day.

*Penicillium* sp. showed the most remarkable radial growth on YESA medium (7.63 cm), followed by CYA (7.2 cm, Fig 2). PDA (6.53 cm) and MEA (5.62 cm) supported moderate growth (Fig 2). In comparison, OA (4.8 cm) and CZA (3.86 cm) had the least expansion (Fig 2). Colonies on YESA were large, radially striated, dark green to bluish-green, surrounded by a yellow peripheral halo, with intense deep orange pigmentation on the reverse (Fig 4), indicating high metabolic activity. CYA supported dense, radially floccose colonies with dark green to grayish-green colonies. In contrast, CZA produced irregular, sparsely sporulating colonies with pale pigmentation (Fig 4). MEA and PDA promoted moderate growth, forming lobed colonies with distinct sporulation patterns. At the same time, OA resulted in thin, sparsely sporulating colonies (Fig 4). These observations highlighted the isolate’s preference for nutrient-rich environments and its potential for use in pigment-production studies related to biocontrol applications. Furthermore, the observed metabolic differences (Figs 2–4) in response to media composition provided significant taxonomic and ecological insights into the isolate’s adaptive strategies.

### 3.3. Micromorphological characteristics of *Aspergillus* sp. and *Penicillium* sp

**Fig 5:**
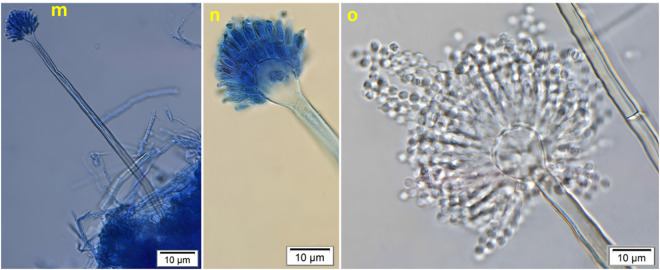
Fungal structures of *Aspergillus* sp. Stipe (m), vesicle, metula and phialide (n), and vesicle and conidia (o).

The conidia of *Aspergillus* sp. ranged from 2.02 to 3.04 μm in diameter (Table 1). The metulae measured between 4.14–7.32 μm in length and 1.75–4.71 μm in diameter (Table 1). Phialides displayed lengths ranging from 3.12 to 7.04 μm and diameters from 1.83 to 4.3 μm (Table 1). The vesicles were globose to subglobose, with lengths ranging from 9.19 to 23.92 μm and diameters from 9.12 to 20.89 μm (Table 1). The stipes were notably long, measuring 442–2092.5 μm in length and 6.57–11.7 μm in diameter (Table 1, Fig 5).

**Table 1:** Dimensional measurements (μm) of fungal structures of *Aspergillus* sp. and *Penicillium* sp.

Conidia Diameter	Metula		Phialide		Vesicle		Stipe		Conidiophore	
	Length	Diameter	Length	Diameter	Length	Diameter	Length	Diameter	Length	Diameter
***Aspergillus* sp.**										
2.02–3.04	4.14–7.32	1.75–4.71	3.12–7.04	1.83–4.3	9.19–23.92	9.12–20.89	442–2092.5	6.57–11.7	–	–
***Penicillium* sp.**										
2.1–3.28	7.26–15.42	2.03–(4.30-8.17)	5.20–8.59	1.23–3.99	–	–	–	–	37.51–334	2.9–5.83

**Fig 6:**
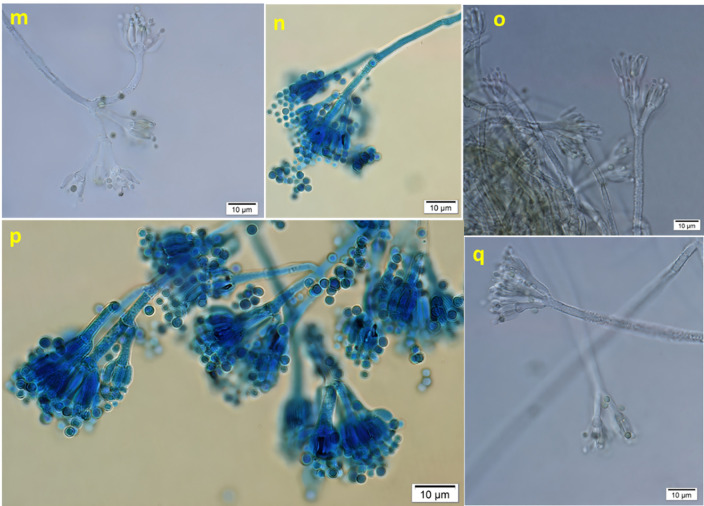
Fungal structures of *Penicillium sp.* Conidiophore, phialide and conidia (m), conidiophore, metula, phialide and conidia (n-q).

Micromorphological features of *Penicillium* sp. were characterized by conidia measuring 2.1 to 3.28 μm in diameter (Table 1). The metulae ranged from 7.26 to 15.42 μm in length and exhibited diameters between 2.03 and a broader range of 4.30 to 8.17 μm (Table 1). Phialides measured 5.20 to 8.59 μm in length with diameters varying from 1.23 to 3.99 μm (Table 1). The conidiophores exhibited considerable variability in size, ranging from 37.51 to 334 μm in length and 2.9 to 5.83 μm in diameter (Table 1, Fig 6).

The observed macro- and micromorphological characteristics of both isolates were consistent with the diagnostic taxonomic criteria and polyphasic identification keys described for *Aspergillus* [37] and *Penicillium* [49] species in previous studies.

### 3.4. Molecular identification of *Aspergillus* sp. and *Penicillium* sp

**Fig 7:**
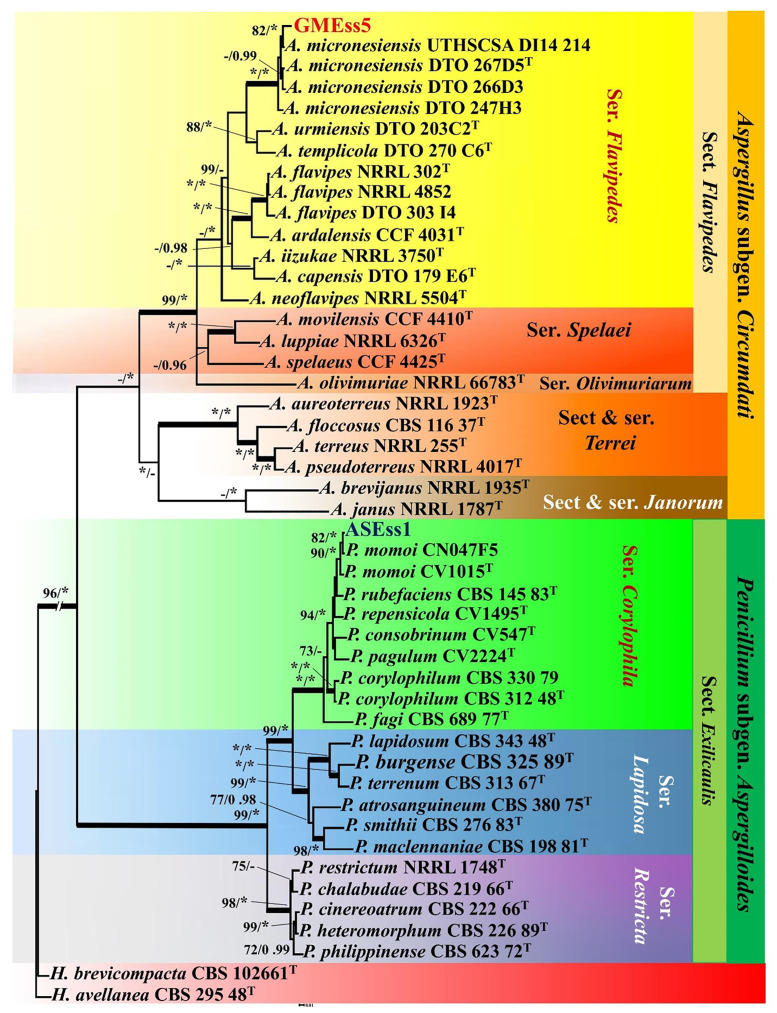
The phylogenetic tree for *Aspergillus* and *Penicillium* species, constructed using Bayesian 50% majority-rule consensus, employed the *ITS*, *TUB*, *CMD*, and *RBP2* gene regions, based on 50 nucleotide sequences under the GTR + G + I model. This tree represents the main phylogenetic relationships for the biocontrol agents investigated in this study. The most recently identified species include GMEss5 and ASEss1. *Hamigera avellanea* (CBS 295 48) and *H. brevicompacta* (CBS 102661) were designated as outgroup taxa. Bayesian posterior probabilities (BPP) exceeding 50% are provided for relevant clades. Ex-type strains are denoted by a superscript T. The Bayesian inference (BI) posterior probability (pp) values and the bootstrap percentages obtained from the maximum likelihood (ML) analysis are illustrated at the nodes, with branches receiving full support being accentuated. Values below 70% bootstrap support (ML) or below 0.70 posterior probability (Bayesian analysis) are represented with a hyphen. The bar indicates the frequency of substitutions per site.”.

Molecular characterization of the examined biocontrol isolates was performed by analyzing four genetic loci: *ITS*, *TUB*, *CMD*, and *RPB2*. Phylogenetic assessment using Bayesian and maximum-likelihood methodologies situated the *Aspergillus* isolate within *A. micronesiensis* clade, exhibiting considerable sequence homology across all genetic markers (S4 Table and Fig 7). In a similar vein, *Penicillium* isolate was found to cluster closely with *P. momoi*, thereby affirming its phylogenetic affiliation (S4 Table and Fig 7). The outgroup species *Hamigera avellanea* and *H. brevicompacta* provided substantial statistical validation (Bayesian posterior probabilities exceeding 50%, S4 Table and Fig 7). These results (Fig 7) reinforced the taxonomic classification of the isolates and suggested potential implications in the fields of plant pathology and biological control.

The partial sequences of the *ITS* rDNA, *TUB*, *CMD*, and *RPB2* obtained from *A. micronesiensis* (*P. momoi*) were deposited in GenBank (NCBI) under the accession numbers PX260295 (PX247873), PX283779 (PX307883), PX283778 **(**PX307883), and PX307882 **(**PX307885), respectively.

### 3.5. Antagonistic effects of *A. micronesiensis* and *P. momoi* against pathogenic *Fusarium* spp

**Fig 8:**
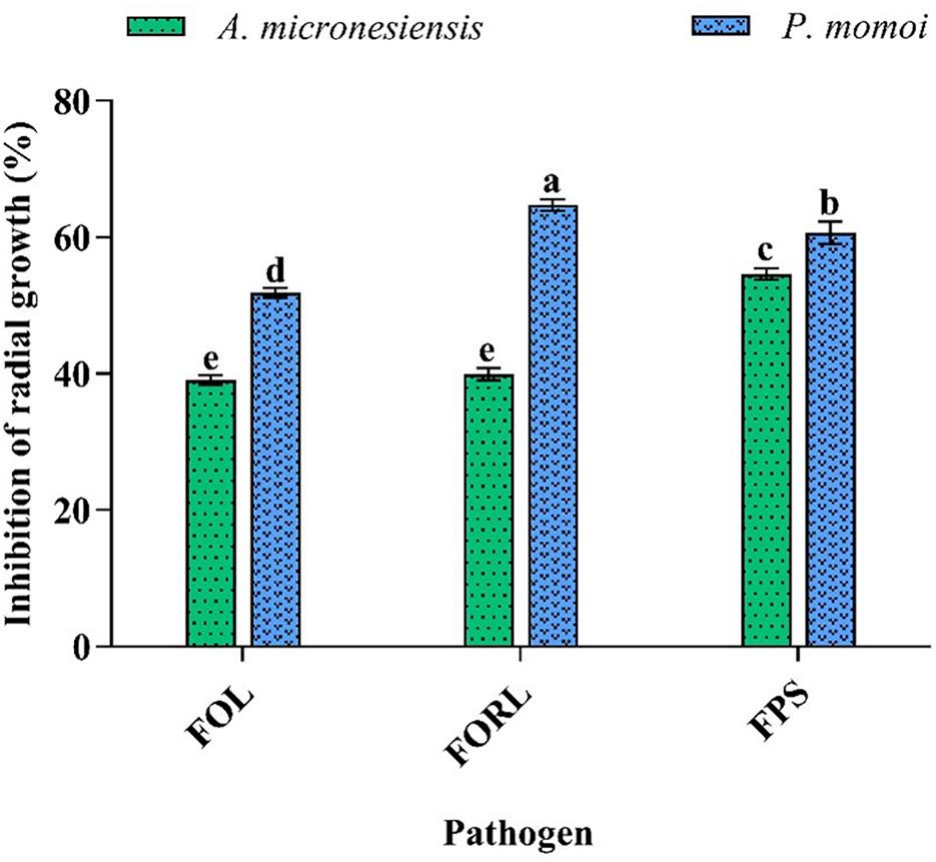
Percentage inhibition of radial growth (PIRG) of the growth of Fusarium oxysporum f. sp. *Lycopersici* (FOL), *F. oxysporum* f. sp. *radicis-lycopersici* (FORL), and *F. pseudograminearum* (FPS) on potato dextrose agar by *Aspergillus micronesiensis* and *Penicillium momoi*. Values represent the mean ± standard error of *n =* 4 independent biological replicates. Significant differences are shown by different letters above the bars (*P* < 0.05).

Antagonistic effects against pathogens were evaluated using dual-culture assays to determine the biocontrol potential of *A. micronesiensis* and *P. momoi* isolates against *F. oxysporum* f. sp. *lycopersici* (FOL), *F. oxysporum* f. sp. *radicis-lycopersici* (FORL) (S4 Fig and Fig 8), and *F*. *pseudograminearum* (FPS, S5 Fig and Fig 8). On PDA, *P. momoi* demonstrated superior antagonistic activity, inhibiting FOL growth by 51.84% (3.30 cm growth compared to 6.85 cm in control) and FORL by 64.74% (2.45 cm versus 6.95 cm in control, Fig 8). *A. micronesiensis* showed moderate suppression of FOL (4.18 cm, 39.05% growth inhibition) and FORL (4.18 cm, 39.93% growth inhibition, Fig 8).

Interestingly, against FPS, *A. micronesiensis* produced a larger inhibition halo (1.06 cm) relative to *P. momoi* (0.15 cm, S5 Fig), although *P. momoi* more effectively restricted FPS growth (3.10 cm, 60.66% growth inhibition, S5 Fig and Fig 8). Notably, *A. micronesiensis* inhibited FPS growth by 54.59% (3.58 cm growth compared to 7.89 cm in control). These observations (S5 Fig) suggested that *P. momoi* primarily exerted antagonism via direct mycelial growth inhibition, whereas *A. micronesiensis* appeared to rely on the production of volatile antifungal metabolites.

### 3.6. Salt tolerance of biocontrol fungi

**Fig 9:**
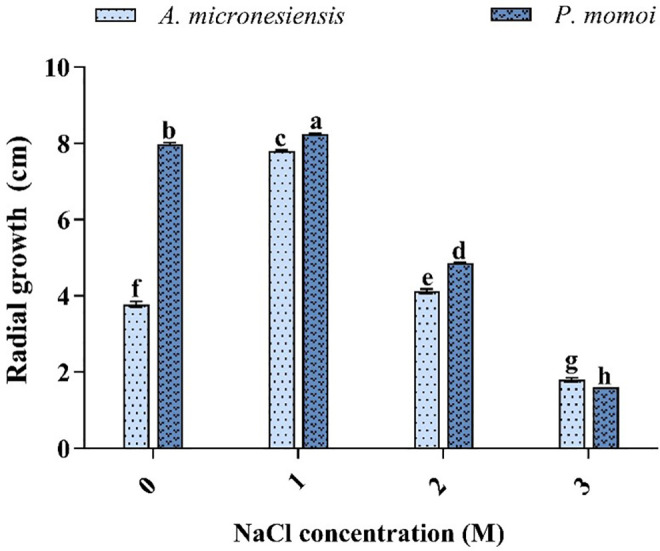
Radial growth of *Aspergillus micronesiensis* and *Penicillium momoi* in culture media containing different concentrations (0, 1, 2, and 3 M) of NaCl. Values represent the mean ± standard error of *n =* 5 independent biological replicates. Significant differences are shown by different letters above the bars (*P* < 0.05).

Halotolerance of the isolates was assessed by culturing them on PDA supplemented with 0, 1, 2, and 3 M NaCl. *A. micronesiensis* exhibited maximum radial growth at 1 M NaCl (7.80 cm), exceeding that of the control (3.78 cm, Fig 9). However, growth markedly declined at higher concentrations (4.12 cm at 2 M and 1.82 cm at 3 M, S6 Fig and Fig 9). *P. momoi* demonstrated vigorous baseline growth at 0 M (7.98 cm), which slightly increased at 1 M (8.24 cm), but progressively decreased at 2 M (4.86 cm) and 3 M (1.6 cm, S7 Fig and Fig 9). These results indicated that moderate salinity enhanced growth in both fungal isolates (*A. micronesiensis* and *P. momoi*), likely due to osmoregulatory mechanisms. In contrast, elevated salinity imposed osmotic stress or ion toxicity. Such responses highlighted the potential of these fungi for applications in saline agroecosystems, although their efficacy diminishes under severe salt stress.

### 3.7. Formulation development and spore germination performance testing

The development of carrier-based formulations was undertaken to maximize spore germination efficiency and ensure the long-term stability of fungal biocontrol isolates. In the case of *A. micronesiensis*, the formulation composed of “wheat bran, sodium alginate, and dipotassium hydrogen phosphate” as well as “wheat bran, sodium alginate, and sodium nitrate” exhibited superior germination performance, followed by the wheat bran–sodium bentonite–sodium nitrate combination (S8 Fig). For *P. momoi*, optimal spore germination was achieved with a formulation containing wheat bran, sodium alginate, and dipotassium hydrogen phosphate, followed by a wheat bran–sodium bentonite–dipotassium hydrogen phosphate formulation (S9 Fig). Based on these outcomes, the optimized carrier formulation (wheat bran, sodium alginate, and dipotassium hydrogen phosphate) was successfully developed for both *A. micronesiensis* and *P. momoi* (S10 Fig), which can be utilized as biofertilizers and biocontrol agents within sustainable agricultural practices.

### 3.8. Study of the stability of selected formulations

**Fig 10:**
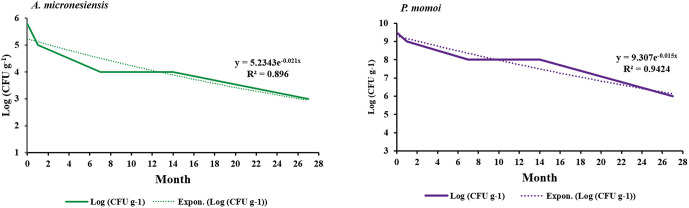
Colony-forming units (CFU) of *Aspergillus micronesiensis* and *Penicillium momoi* in wheat bran + sodium bentonite-based powder and tablet formulations over 27 months.

The optimized formulations were evaluated for their ability to maintain the population of existing antagonists over time. Stability evaluations of the formulations over a 27-month storage period demonstrated that “wheat bran, sodium alginate, and dipotassium hydrogen phosphate” formulation maintained high levels of viable spores for both species (*A. micronesiensis* and *P. momoi*, Fig 10). In *A. micronesiensis*, counts decreased from 6 × 10^5^ to 1 × 10^3^ spores g^-1^ formulation, whereas in *P. momoi*, they declined from 3 × 10^9^ to 1 × 10^6^ spores g^-1^ formulation. These findings confirmed that the selected carrier systems constituted the most suitable formulations, ensuring extended viability and stability of the fungal isolates for practical field applications.

### 3.9. Greenhouse evaluation of optimized formulation of biocontrol agents under combined drought and pathogen stress

Analysis of variance (ANOVA) revealed that cultivar, drought stress, pathogen inoculation, and biocontrol/chemical treatments significantly affected the evaluated traits, including SPAD value, plant height, shoot and root biomass, and disease score (S6 Table). For SPAD, all interaction effects were significant except the cultivar × drought stress × pathogen inoculation interaction. Plant height was significantly influenced by most interaction effects, whereas the cultivar × drought stress and drought stress × pathogen inoculation × biocontrol/chemical treatment interactions were not significant (S6 Table). Shoot fresh weight (SFW) and root dry weight (RDW) were significantly affected by most interaction effects, while the cultivar × pathogen inoculation interaction was not significant (S6 Table). Root fresh weight (RFW) and shoot dry weight (SDW) were significantly influenced by all interaction terms except for the four-way interaction effect observed for RFW (S6 Table). Disease score was significantly affected by the cultivar × pathogen inoculation, drought stress × pathogen inoculation, and pathogen inoculation × biocontrol/chemical treatment interactions (S6 Table), indicating differential disease responses depending on host genotype, irrigation regime (drought stress), and biological/chemical treatment type.

The highest SPAD values were recorded in drought-stressed, non-inoculated plants treated with the biological formulations, reaching 46.6 and 46.7 in Hashtrood-PenF and Sadra-AspF, respectively, representing increases of 11.2% and 28.2% compared with the corresponding controls (Table 2). Under *F. pseudograminearum* stress, PenF improved SPAD value in Hashtrood (26.7%) and Sadra (15.7%) cultivars compared with their respective controls (Table 2).

**Table 2:** Effects of drought stress (-/+), *Fusarium pseudograminearum* (-/+), and biocontrol/chemical treatments on plant height (cm), shoot fresh weight (SFW, g), root fresh weight (RFW, g), shoot dry weight (SDW, g), root dry weight (RDW, g), and disease score of two wheat cultivars (Hashtrood and Sadra).

Treatment	*F. pseudograminearum* ^-^							*F. pseudograminearum* ^+^						
**Spad**	**Height**	**SFW**	**RFW**	**SDW**	**RDW**	**DS**	**Spad**	**Height**	**SFW**	**RFW**	**SDW**	**RDW**	**DS**
**Hashtrood-Drought** ^**-**^														
AspF	41.0e	46.8f	6.1b	5.8a	0.84a	0.548c	0.0h	35.8klm	42.0j	3.7h	3.5jk	0.52h	0.415i	2.0d
PenF	38.7g	47.5e	6.3a	6.0a	0.75c	0.499f	0.0h	37.0j	40.5l	3.7h	3.3lm	0.41l	0.327m	1.8e
EmpF	35.8klm	48.0d	4.1f	4.1h	0.56g	0.416i	0.0h	29.2v	36.2n	2.4m	2.4op	0.38mn	0.248s	3.2a
EmpF + TEB	32.5p	41.5k	3.8h	4.8d	0.47j	0.520d	0.0h	31.6stu	36.2n	3.4i	3.0n	0.26tu	0.224t	3b
TEB	35.0n	45.5g	4.2e	4.3f	0.55g	0.448g	0.0h	33.4o	42.5i	4.1ef	3.5j	0.44k	0.384j	3b
**Hashtrood-Drought** ^**+**^														
AspF	42.5c	33.2q	2.6l	2.4o	0.42kl	0.225t	0.0h	36.9j	32.5r	2.1n	1.8s	0.30p	0.193v	1.8e
PenF	46.4a	35.5o	2.8k	3.4kl	0.38m	0.359k	0.0h	37. 8hi	32.0s	2.1n	2.1q	0.28qrs	0.265pq	2.2c
EmpF	36.2k	30.0u	1.9o	2.1q	0.30p	0.260qr	0.0h	32.1qr	24.2B	1.5q	1.3v	0.20w	0.161y	3.2a
EmpF + TEB	38.0h	27.8x	2.1n	2.3op	0.27ts	0.268op	0.0h	33.4o	24.7A	1.3r	1.6t	0.18x	0.170x	3.0b
TEB	39.8f	29.0vw	1.9o	2.0q	0.29pq	0.252s	0.0h	32.0qrs	25.5z	2.6l	2.1q	0.246uv	0.206u	3.2a
**Sadra-Drought** ^**-**^														
AspF	40.2f	51.0a	6.3a	4.7e	0.81b	0.624a	0.0h	35.6lm	49.8b	5.6c	3.5jk	0.64e	0.426h	0.8g
PenF	37.6i	51.0a	6.0b	5.2c	0.83ab	0.562b	0.0h	36.1k	48.5c	4.4d	4.3fg	0.72d	0.519d	0.8g
EmpF	36.0kl	45.2g	4.5d	4.2g	0.59n	0.510e	0.0h	31.2u	42.5i	3.2j	3.9i	0.33o	0.338l	1.5f
EmpF + TEB	36.0kl	45.5g	4.2ef	4.0hi	0.47j	0.495f	0.0h	32.2pq	41.8jk	3.4i	3.2m	0.50i	0.360k	1.5f
TEB	35.5m	44.8h	3.9g	4.0hi	0.50i	0.454g	0.0h	31.4tu	40.5l	3.8h	3.4jk	0.47j	0.378j	2.2c
**Sadra-Drought** ^**+**^														
AspF	46.7a	36.2n	2.9k	1.96r	0.47j	0.253rs	0.0h	39.0g	35.2o	1.0t	1.5u	0.288pqr	0.205u	0.8g
PenF	45.0b	38.0m	2.3m	2.3op	0.36n	0.344l	0.0h	37.9hi	34.2p	1.9o	1.8s	0.263tsu	0.192v	0.8g
EmpF	42.0d	32.2rs	1.9o	2.27p	0.26tsu	0.306n	0.0h	34.8n	27.2y	0.7u	1.1w	0.13y	0.166xy	2.2c
EmpF + TEB	40.2f	30.0u	1.3r	2.1q	0.20w	0.207u	0.0h	33.6o	28.8w	1.0t	1.0w	0.23v	0.1360z	2.2c
TEB	39.8f	31.5t	1.7p	2.4op	0.27rst	0.273o	0.0h	31.7rst	29.2v	1.1s	1.4v	0.19wx	0.178w	3.0b

Values represent the mean of 𝑛 = 4 independent biological replicates. Means within a column followed by the same letter are not significantly different (p ≤ 0.05).

Drought^-^; non-drought conditions, drought^+^; drought stress conditions, *F. pseudograminearum*^-^; non-inpculated plants, *F. pseudograminearum*^+^; *F. pseudograminearum*-inoculated plants

The greatest plant height was observed in Sadra cultivar under non-stress conditions treated with AspF or PenF, reaching 51.0 cm, which was approximately 12.8% higher than the corresponding control (EmpF, Table 2). Under drought, *F. pseudograminearum* inoculation, and combined stress conditions, biological formulations (AspF or PenF) improved plant height in both cultivars compared with their respective controls (Table 2). Increases in plant height under combined drought and pathogen stress reached 34.3% in Hashtrood and 29.4% in Sadra (Table 2).

Drought stress and *F. pseudograminearum* inoculation markedly reduced shoot and root biomass traits (Table 2). However, application of the biological formulations generally alleviated these negative effects across both cultivars. Among the tested combinations, the highest SFW values were observed in Hashtrood-PenF and Sadra-AspF treatments (6.3 g; Table 2). Hashtrood cultivar also exhibited the greatest RFW under PenF and AspF treatments, reaching 6.0 and 5.8 g, respectively. Under *F. pseudograminearum* stress, RDW of Sadra cultivar treated with PenF reached 0.519 g, representing an increase of approximately 53.6% compared with the corresponding control (EmpF; Table 2), highlighting the positive effect of the biocontrol formulation on root development under disease pressure.

As shown in Table 2, biological formulations (AspF and PenF) alleviated disease score in *F. pseudograminearum*-inoculated plants compared with the corresponding controls. In the absence of pathogen inoculation, disease score was zero in all treatments (Table 2). In Hashtrood, the lowest disease score was observed under pathogen stress alone and combined drought–pathogen stress, with PenF (1.8) and AspF (1.8), respectively, representing a 43.8% reduction compared with the corresponding infected controls (Table 2). In Sadra, *F. pseudograminearum*-inoculated plants treated with biological formulations (AspF and PenF) under both non-drought and drought conditions showed the lowest disease score (0.8), which was 46.7% and 63.6% lower than the corresponding controls, respectively (Table 2).

Overall, the microbial formulations, AspF and PenF, were more effective than the control (EmpF) and chemical fungicide treatment (TEB) in mitigating the adverse effects of drought and *F. pseudograminearum* stress on plant growth and disease development (Table 2).

## 4. Discussion

This study systematically evaluated *A. micronesiensis* and *P. momoi* as endophytic biocontrol agents against Fusarium pathogens, integrating morphological, molecular, and functional analyses. The observed variability in colony development across different culture media (Figs 2-4) reflected metabolic plasticity and adaptive growth strategies, suggesting ecological versatility that may contribute to environmental persistence rather than serving solely as a taxonomic feature.

Micromorphological characterization, including conidial size, metulae, phialides, vesicles, and stipe measurements (Table 1 and Figs 5 and 6), provided additional taxonomic resolution. Integration of morphological identification with multilocus molecular analysis (*ITS*, *β-tubulin*, *calmodulin*, and *RPB2*; Fig 7) confirmed the phylogenetic positions of *A. micronesiensis* and *P. momoi*, supporting the reliability of combining morphological and molecular approaches for identification of cryptic fungal taxa.

Antagonistic assays indicated complementary mechanisms of pathogen suppression. *P. momoi* likely inhibited *Fusarium* growth through direct mycelial competition, whereas *A. micronesiensis* appeared to rely on diffusible metabolites (S4, S5 Fig, and Fig 8). Beyond direct mycelial inhibition and antibiosis, the observed complementary antagonistic activity may also involve the induction of systemic resistance in the host plant, as previously reported in endophyte-mediated biocontrol systems [50,51]. These functional differences suggest that co-application of both fungi may enhance biocontrol effectiveness [25,52].

Moderate salinity (1 M NaCl) enhanced fungal growth (S6, S7 Figs, and Fig 9), indicating the presence of adaptive osmoregulatory mechanisms, whereas higher salinity imposed physiological stress. Such tolerance patterns may be associated with fungal osmoregulatory mechanisms, including the accumulation of compatible solutes (e.g., glycerol, trehalose) and ion homeostasis regulation, highlighting the potential application of these isolates in saline-affected agroecosystems while suggesting careful consideration of environmental constraints [53].

Although salinity and drought are distinct environmental stresses, both impose osmotic constraints that limit water availability and trigger related adaptive responses. Therefore, the *in vitro* salinity assay was used as a preliminary indicator of the ability of the fungal isolates to tolerate osmotic stress. The greenhouse experiment demonstrated that drought stress and *F. pseudograminearum* infection resulted in a marked reduction in plant growth and physiological traits, including SPAD, plant height, and biomass production. However, application of the endophytic fungal formulations improved plant performance under stress conditions compared with the corresponding controls (Table 2). Overall, the results confirmed that the formulated endophytes were able to partially alleviate the negative effects of the abiotic and biotic stress under greenhouse conditions.

Formulation studies indicated that wheat bran–sodium alginate–dipotassium hydrogen phosphate matrices supported spore viability and long-term storage stability for up to 27 months (S8–S10 Fig and Fig 10). These findings suggest improved shelf stability of biocontrol formulations; however, further field-scale validation is required to confirm their performance under practical agricultural conditions.

A key limitation of this study is the lack of mechanistic resolution at the metabolite and molecular levels. Although antagonistic activity and salt tolerance were demonstrated under controlled conditions, the specific bioactive compounds responsible for antifungal effects, as well as the osmolytes and metabolic pathways involved in salinity adaptation, were not characterized. In addition, while preliminary greenhouse evaluations provide partial *in planta* support, the findings remain primarily based on controlled experimental conditions. Therefore, extrapolation to field environments should be made cautiously, as complex plant–microbe–environment interactions may significantly influence the performance of these endophytic fungi. Future studies employing metabolomics profiling and targeted chemical analyses are essential to elucidate the underlying mechanisms and strengthen the functional interpretation of these biocontrol agents.

## 5. Conclusion

This study demonstrates the potential of *A. micronesiensis* and *P. momoi* as effective biocontrol agents against key *Fusarium* pathogens. Both fungi exhibited strong and complementary antagonistic activity through direct mycelial inhibition and metabolite-mediated suppression under *in vitro* conditions.

The isolates also showed moderate halotolerance in culture-based assays, indicating their ability to grow under saline conditions *in vitro*. Carrier-based formulations maintained high spore viability and long-term stability, supporting their potential for further development.

Preliminary greenhouse experiments further demonstrated that application of the fungal formulations improved plant growth performance and reduced disease score under stress conditions compared with controls (EmpF and TEB).

However, it is important to emphasize that fungal responses to salinity were evaluated only under *in vitro* conditions, and plant-level physiological mechanisms of salt stress mitigation were not directly investigated in this study. Therefore, future research should include field-scale validation, detailed physiological analyses, and metabolomics characterization to fully elucidate the biocontrol and stress-related effects of these endophytic fungi.

Collectively, these findings support the potential application of these endophytic fungi in sustainable disease management and integrated pest management strategies under stress-prone agricultural systems.

**Highlights**

- Both species of *Aspergillus micronesiensis* and *Penicillium momoi* strongly inhibited key Fusarium pathogens.
- These endophytic fungi had complementary biocontrol: direct inhibition and antibiosis.
- *A. micronesiensis* and *P. momoi* tolerate high salinity, growing in up to 3 M NaCl.
- Biological formulations maintain spore viability for over 27 months.
- Dual biocontrol and osmotic stress mitigation support sustainable crop management.

## Supporting information

S1 Table Primers used for amplification and sequencing. (DOCX)

S2 Table Media used for morphological characterization. (DOCX)

S3 Table Accession numbers used for phylogenetic analysis of *Fusarium pseudograminearum.* (DOCX)

S4 Table Accession numbers used for phylogenetic analysis of *Aspergillus* sp. and *Penicillium* sp. (DOCX)

S5 Table Different compositions of carrier and stabilizers for maximum spore germination. (DOCX)

S6 Table Analysis of variance for the effects of wheat cultivar, soil moisture content, pathogen and formulations on SPAD value, plant height, shoot and root biomass, and disease score. (DOCX)

S1 Fig *Fusarium oxysporum* f. sp. *lycopersici* (FOL) and *F. o.* f. sp. *radicis-lycopersici* (FORL) on potato dextrose agar. (PNG)

S2 Fig PCR analysis of polygalacturonase gene region of *Fusarium oxysporum* f. sp. *lycopersici* with uni primer and in 2% agarose gel. Ladder DNA 1 Kbp (a), DNA fragments (670–672 bp) of two isolates amplified with primer pair uni (b and c).
(TIF)

S3 Fig PCR analysis of polygalacturonase gene region of *Fusarium oxysporum* f. sp. *lycopersici* with Spr1 primer and in 2% agarose gel. Ladder DNA 1 Kbp (a), DNA fragments (700 bp) of one isolate amplified with primer pair Spr1 (b).
(PNG)

S4 Fig Dual culture assays of biocontrol isolates against pathogenic fungi. *Aspergillus micronesiensis* against *Fusarium oxysporum* f. sp. *lycopersici* (FOL, a). *Penicillium momoi* against FOL (b). *A. micronesiensis* against *F. oxysporum* f. sp. *radicis-lycopersici* (FORL, c). *P. momoi* against FORL (d).
(PNG)

S5 Fig *Fusarium pseudograminearum* (FPS; A1 and A2), Dual cultures of biocontrol isolates with pathogenic fungi: *Aspergillus micronesiensis* against FPS (B1 and B2), *Penicillium momoi* against FPS (C1 and C2). Indices 1 and 2 correspond to the top and bottom views, respectively.
(PNG)

S6 Fig *Aspergillus micronesiensis* in culture media containing different concentrations (0, 1, 2, and 3 M) of NaCl. (PNG)

S7 Fig *Penicillium momoi* in culture media containing different concentrations (0, 1, 2, and 3 M) of NaCl. (PNG)

S8 Fig Number of germinated spores in different formulations of *Aspergillus micronesiensis.* The materials used in this study included wheat bran (Wb), sodium alginate (Sa), sodium bentonite (Sb), talc (Ta), sodium nitrate (Sn), and dipotassium hydrogen phosphate (Dhp).
(TIF)

S9 Fig Number of germinated spores in different formulations of *Penicillium momoi.* The materials used in this study included wheat bran (Wb), sodium alginate (Sa), sodium bentonite (Sb), talc (Ta), sodium nitrate (Sn), and dipotassium hydrogen phosphate (Dhp).
(TIF)

S10 Fig Proposed formulation for *Penicillium momoi* and *Aspergillus micronesiensis* based on wheat bran + sodium alginate and the ratio of its components. (TIF)
