# Supplementary material for: Dual biocontrol and osmotic stress mitigation by endophytic Aspergillus micronesiensis and Penicillium momoi against fusarium pathogens
Source: PLoS One. 2026 Jul 29;21(7):e0353217. doi: 10.1371/journal.pone.0353217 (PMC13421755; doi:10.1371/journal.pone.0353217)
Supplement: S1 Table — (DOCX) [file pone.0353217.s001.docx]

| **S1Table** | | | |
| --- | --- | --- | --- |
| **Locus** | **Primer name** | **Primer sequencing(5´– 3´)** | **Reference** |
| Internal Transcribed Spacer | ITS1 (F) | TCCGTAGGTGAACCTGCGG | [33] |
|  | ITS4 (R) | TCCTCCGCTTATTGATATGC |  |
| β-tubulin | Bt_2_a (F) | GGTAACCAAATCGGTGCTGCTTTC | [34] |
|  | Bt_2_b (R) | ACCCTCAGTGTAGTGACCCTTGGC |  |
| RNA Polymerase II second largest subunit | 5F (F) | GAYGAYMGWGATCAYTTYGG | [38] |
|  | 7CR (R) | CCCATRGCTTGYTTRCCCAT |  |
| Calmodulin | CMD5 (F) | CCGAGTACAAGGARGCCTTC | [39] |
|  | CMD6 (R) | CCGATRGAGGTCATRACGTGG |  |
| Uni | Uni f | ATCATCTTGTGCCAACTTCAG | [35] |
|  | Uni r | GTTTGTGATCTTTGAGTTGCCA |  |
| sprl | sprl f | GGGCTCCTAATCCGTGCTTCA | [35] |
|  | sprl r | GGTGGAGGATCGGGTTTGTTTC |  |
