# Supplementary material for: Dual biocontrol and osmotic stress mitigation by endophytic Aspergillus micronesiensis and Penicillium momoi against fusarium pathogens
Source: PLoS One. 2026 Jul 29;21(7):e0353217. doi: 10.1371/journal.pone.0353217 (PMC13421755; doi:10.1371/journal.pone.0353217)
Supplement: S2 Table — (DOCX) [file pone.0353217.s002.docx]

| **S2Table** | | | |
| --- | --- | --- | --- |
| **Czapek Stock Solution (100 ml) (Pitt 1979)** | | **Malt Extract Agar (MEA, Samson et al. 2010)** | |
| NaNO₃ | 30 g | Malt extract (Oxoid CM0059) | 50 g |
| KCl | 5 g | CuSO₄·5H₂O | 0.005 g |
| MgSO₄·7H₂O | 5 g | ZnSO₄·7H₂O | 0.01 g |
| FeSO₄·7H₂O | 0.1 g | dH₂O | 1000 ml |
| dH₂O | 100 ml | Autoclave at 121 °C for 25 min, pH 5.4 ± 0.2 | |
| Store at 4–10 °C | | **Oatmeal Agar (OA, Samson et al. 2010)** | |
| **Trace Elements Stock Solution (100 ml)** | |  |  |
| CuSO₄·5H₂O | 0.5 g | Oatmeal flakes | 30 g |
| ZnSO₄·7H₂O | 0.1 g | CuSO₄·5H₂O | 0.005 g |
| dH₂O | 100 ml | ZnSO₄·7H₂O | 0.01 g |
| Store at 4–10 °C |  | Agar | 20 g |
| **Czapek's Agar (CZ, Raper & Thom 1949)** | | dH₂O | 1000 ml |
|  |  | Autoclave at 121 °C for 25 min, pH 6.5 ± 0.2 | |
| Czapek concentrate | 10 ml | **Yeast Extract Sucrose Agar (YES, Frisvad 1981)** | |
| Sucrose | 30 g |  |  |
| CuSO₄·5H₂O | 0.005 g | Yeast extract (Difco) | 20 g |
| ZnSO₄·7H₂O | 0.001 g | Sucrose | 150 g |
| Agar | 20 g | MgSO₄·7H₂O | 0.5 g |
| dH₂O | 1000 ml | CuSO₄·5H₂O | 0.005 g |
| Autoclave at 121 °C for 15 min | | ZnSO₄·7H₂O | 0.001 g |
| **Czapek Yeast Autolysate Agar (CYA, Pitt 1979)** | | Agar | 20 g |
|  |  | dH₂O | 885 ml |
| Czapek concentrate | 10 ml | Autoclave at 121 °C for 25 min, pH 6.5 ± 0.2 | |
| Sucrose | 30 g | **Potato Dextrose Agar (PDA)** | |
| Yeast extract (Difco) | 5 g |  |  |
| K₂HPO₄ | 1 g | Dextrose | 20 g |
| CuSO₄·5H₂O | 0.005 g | Agar | 20 g |
| ZnSO₄·7H₂O | 0.01 g | dH2O | 1000 ml |
| Agar | 20 g | Mix well and autoclave at 121°C for 25 min. | |
| dH₂O | 1000 ml |  |  |
| Autoclave at 121 °C for 25 min. pH 6.2 ± 0.2 | |  |  |
