# Supplementary material for: Dual biocontrol and osmotic stress mitigation by endophytic Aspergillus micronesiensis and Penicillium momoi against fusarium pathogens
Source: PLoS One. 2026 Jul 29;21(7):e0353217. doi: 10.1371/journal.pone.0353217 (PMC13421755; doi:10.1371/journal.pone.0353217)
Supplement: S3 Table — (DOCX) [file pone.0353217.s003.docx]

| **S3Table** | | | | | |
| --- | --- | --- | --- | --- | --- |
|  |  | |  | **GenBank accession numbers** | |
| **Species name** | **Collection numbers** | | **Location, Substrate** | **ITS** | **BenA** |
| ***Fusarium pseudograminearum*** | | **F01** | **Iran(West Azerbaijan- Shahin Dezh County) - Wheat** | **PX472025** | **PX516788** |
| *Fusarium pseudograminearum* | | NRRL 28062^T^ | New South Wales, Australia, Darling downs- Barley crown | NR_182316 | AF107867 |
| *Fusarium pseudograminearum* | | WZ-2B | Henan, China – From Wheat | JN862234 | JN862231 |
| *Fusarium pseudograminearum* | | RPYQF-25 | China: Henan,puyang - Arachis hypogaea | PV596285 | PV631041 |
| *Fusarium cerealis* | | NRRL 25491 | Netherlands-Iris hollandica | AF006340 | AF212782 |
| *Fusarium robustum* | | NRRL 13392^T^ | Argentina - Parana´ pine (*Araucaria angustifolia*) | U85539 | U85573 |
| *F. lunulosporum* | | NRRL 13393 = BBA 62459 = CBS 636.76 = FRC R-5822 = IMI 322097^T^ | South Africa - *Citrus paradisi* | MH861017 | PQ274011 |
| *Fusarium boothii* | | NRRL 29011 | South Africa - | NR_121203 | AF212762 |
| *Fusarium culmorum* | | 1067 | China: Suzhou - *Glycine max*, source=root | PQ814365 | - |
| *Fusarium culmorum* | | CBS 250.52 | Rye | DQ453702 | - |
| *Fusarium culmorum* | | CBS 122.73 | UK, *Triticum* | DQ453703 | - |
| *Fusarium nelsonii* | | NRRL 13338 | USA - | GQ505434 | - |
| *Fusarium nelsonii* | | CIB04 | Fruit of cucumber in Guangzhou, China | MN117676 | - |
| **The sequences studied in this research are shown in bold.** | | |  |  |  |
