# Supplementary material for: Dual biocontrol and osmotic stress mitigation by endophytic Aspergillus micronesiensis and Penicillium momoi against fusarium pathogens
Source: PLoS One. 2026 Jul 29;21(7):e0353217. doi: 10.1371/journal.pone.0353217 (PMC13421755; doi:10.1371/journal.pone.0353217)
Supplement: S4 Table — (DOCX) [file pone.0353217.s004.docx]

| **S4Table** | | | | | | | |
| --- | --- | --- | --- | --- | --- | --- | --- |
|  |  |  | **GenBank accession numbers** | | | |  |
| **Species name** | **Collection numbers** |  | **ITS** | **BenA** | **CaM** | **RPB2** |  |
| ***Aspergillus micronesiensis*** | **GMEss5** |  | **PX260295** | **PX283779** | **PX283778** | **PX307882** |  |
| *A. micronesiensis* | DTO 267-D5 = CBS 138183 |  | KJ775548 | KJ775085 | KP987067 | KP987023 |  |
| *A. micronesiensis* | UTHSCSA DI14-214 |  | LT899480 | LT899529 | LT899582 | LT899637 |  |
| *A. micronesiensis* | DTO 266-D3 |  | KP987080 | KP987048 | KP987063 | KP987037 |  |
| *A. micronesiensis* | CBS 147045=DTO 247-H3 |  | KP987079 | KP987047 | KP987062 | KP987036 |  |
| *A. flavipes* | NRRL 302 = CCF 3067 = IMI 171885 = ATCC 24487= FRR 0302 |  | EF669591 | EU014085 | EF669549 | EF669633 |  |
| *A. flavipes* | DTO 303-I4 = NRRL 4852 = CCF 4836 = IMI 345934 |  | KP987083 | KP987053 | KP987070 | KP987019 |  |
| *A. flavipes* | NRRL 4852 = IMI 345934 = CCF 4836 (ex- type of A. archiflavipes |  | LM999909 | LM644261 | LM644241 | LM644260 |  |
| *A. neoflavipes* | NNRL 5504 = ATCC 24484 = CBS 260.73 = IMI 171883 = IFM 40894 = CCF 4552 |  | EF669614 | EU014084 | EF669572 | EF669656 |  |
| *A. iizukae* | NRRL 3750 = CBS 541.69 = IMI 141552 = CCF 4548 |  | EF669597 | EU014086 | EF669555 | EF669639 |  |
| *A. ardalensis* | CCF 4031 = NRRL 62824 = CCF 4426= CMF ISB 1688= CBS 134372 |  | FR733808 | HG916683 | HG916725 | HG916704 |  |
| *A. movilensis* | CCF 4410 = NRRL 62819 = CMF ISB 2614 = CBS 134395 |  | HG915904 | HG916697 | HG916740 | HG916718 |  |
| *A. spelaeus* | CCF 4425 = CMF ISB 2615 = CBS 134371 = NRRL 62826 |  | HG915905 | HG916698 | HG916741 | HG916719 |  |
| *A. brevijanus* | NRRL 1935 = CBS 111.46 = ATCC 16828 = CBS 119.45 = IMI 016066ii = IMI 16066 = NCTC 6971 = QM 7417 = WB 1935 |  | EF669582 | EU014078 | EF669540 | EF669624 |  |
| *A. janus* | NRRL 1787 = CBS 118.45 = IMI 16065 = NCTC 6970 |  | EF669578 | EU014076 | EF669536 | EF669620 |  |
| *A. floccosus* | CBS 116.37 = IBT 10846 = WB 4872 = IBT 22556 |  | KP987086 | FJ491714 | KP987066 | KP987021 |  |
| *A. aureoterreus* | NRRL 1923 = CBS 503.65 = ATCC 16793 = IFO 30536 = IMI 82431 = MUCL 38644 = QM 7472 = VKM F-2035 = WB 1923 |  | EF669580 | EF669524 | EF669538 | EF669622 |  |
| *A. terreus* | NRRL 255 = CBS 601.65 = ATCC 10071 = ATCC 1012 = IFO 33026 = IMI 017294ii = IMI 17294 = JCM 10257 = LSHBA c .24 = MUCL 38640 = NCTC 981 = NRRL 543 = QM 1 = QM 1991 = Thom 144 = VKMF-67 = WB 255 |  | EF669586 | EF669519 | EF669544 | EF669628 |  |
| *A. pseudoterreus* | NRRL 4017=CBS 123890 |  | EF669598 | EF669523 | EF669556 | EF669640 |  |
| *A. luppiae* | NRRL 6326 = CBS 653.74 = CCF 4545 |  | EF669617 | EU014079 | EF669575 | EF669659 |  |
| *A. templicola* | DTO 270-C6T= CBS 138181 |  | KJ775545 | KJ775092 | KJ775394 | KP987017 |  |
| *A. urmiensis* | DTO 203-C2 =CCTU 742 = CBS 139558 = IBT 32593 |  | KP987073 | KP987041 | KP987056 | KP987030 |  |
| *A._capensis* | DTO 179-E6 = CBS 138188 |  | KJ775550 | KJ775072 | KJ775279 | KP987020 |  |
| *A. olivimuriae* | NRRL 66783 = CCF 6208 |  | NR_165521 | MH492010 | MH492011 | MH492012 |  |
| ***Penicillium momoi*** | **ASEss1** |  | **PX247873** | **PX307884** | **PX307883** | **PX307885** |  |
| *P. momoi* | CBS 139157 = DAOM 241077 = DTO 182-G4 = CV 1015 |  | JX140895 | JX141073 | JX157479 | KP064673 |  |
| *P. momoi* | CN047F5 |  | ----- | OR241734 | OR241823 | OR241878 |  |
| *P. corylophilum* | CBS 312.48 = TCC9784 = ATHUM2890 = CECT 2270 = FRR 802 = IMI 039754 = MUCL 28671 = MUCL 29073 = MUCL 29131 = NRRL 802 = QM 7510 |  | AF033450 | JX141042 | KP016780 | KP064631 |  |
| *P. corylophilum* | CBS 330.79 = IJFM 5147 |  | GU944557 | GU944519 | GU944607 | JN406569 |  |
| *P. lapidosum* | CBS 343.48 = ATCC 10462 = CCT4477 = IFO 6100 = IMI 039743 = NRRL 718 = QM 1928 |  | MN431392 | KJ834465 | FJ530984 | JN121500 |  |
| *P. maclennaniae* | CBS 198.81 = DAR 35238 |  | KC411689 | KJ834468 | KP016791 | KP064648 |  |
| *P. atrosanguineum* | CBS 380.75 = FRR 1726 = IMI 197488 |  | JN617706 | KJ834435 | KP016771 | JN406557 |  |
| *P. burgense* | CBS 325.89 |  | KC411736 | KJ834437 | KP016772 | JN406572 |  |
| *P. chalabudae* | CBS 219.66 = ATCC 18322 = ATCC 18329 = FRR 3393 = VKMF-1037 |  | KP016811 | KP016748 | KP016767 | KP064572 |  |
| *P. cinereoatrum* | CBS 222.66 = ATCC 22350 = FRR 3390 = IJFM 5024 = IMI 113676 = VKMF-856 |  | KC411700 | KJ834442 | KP125335 | JN406608 |  |
| *P. consobrinum* | CBS 139144 = DAOM 241072 = DTO 181-H9 = CV 547 |  | JX140888 | JX141135 | JX157453 | KP064619 |  |
| *P. fagi* | CBS 689.77 = CCMF-696 = IJFM 3049 = IMI 253806 = VKMF-2178 |  | MH861113 | KJ834449 | KP016784 | JN406540 |  |
| *Penicillium restrictum* | NRRL 1748 = ATCC 11257 = FRR 1748 = IMI 040228 = CBS 367.48 = QM 1962 |  | AF033457 | KJ834486 | KP016803 | JN121506 |  |
| *P. heteromorphum* | CBS 226.89 |  | KC411702 | KJ834455 | KP016786 | JN406605 |  |
| *P. philippinense* | CBS 623.72 = FRR 1532 = NHL 6130 |  | KC411770 | KJ834482 | KP016799 | JN406543 |  |
| *P. repensicola* | CBS 139160 = DAOM 241080 = DTO 183-B8 = CV 1495 |  | JX140893 | JX141150 | JX157490 | KP064660 |  |
| *P. rubefaciens* | CBS 145.83 = CECT 2752 |  | KC411677 | KJ834487 | KP016804 | JN406627 |  |
| *P. smithii* | CBS 276.83 = CECT 2744 = IMI 259693 |  | KC411723 | KJ834492 | KP016806 | JN406589 |  |
| *P. terrenum* | CBS 313.67 = ATCC 18547 = CSIR 1022 = IMI 136208 |  | MH858978 | KJ834496 | KP016808 | JN406577 |  |
| *P. pagulum* | CBS 139166 = DAOM 241069 = DTO 183-H2 = CV 2224 |  | JX140898 | JX141070 | JX157519 | KP064655 |  |
| *Hamigera_avellanea* | CBS 29548T = ATCC 10414 = CECT 2265 = DSM 2208 = IMI 040230 = NRRL 1938 |  | LC076687 | LC076692 | LC076690 | JF417424 |  |
| *H. brevicompacta* | CBS 102661T= AS 3.4676 |  | MN431402 | MN969421 | MN969342 | MN969203 |  |
| The sequences studied in this research are shown in bold. | | | | | | | |
