# Supplementary material for: Dual biocontrol and osmotic stress mitigation by endophytic Aspergillus micronesiensis and Penicillium momoi against fusarium pathogens
Source: PLoS One. 2026 Jul 29;21(7):e0353217. doi: 10.1371/journal.pone.0353217 (PMC13421755; doi:10.1371/journal.pone.0353217)
Supplement: S5 Table — (DOCX) [file pone.0353217.s005.docx]

| **S5Table** | | | |
| --- | --- | --- | --- |
| **Suspensibility** | **Wettability** | **Stabilizers** | **Carriers** |
| CMC | Tween 20 | Sn + Dhp | Wb |
| CMC | Tween 20 | Sn | Wb |
| CMC | Tween 20 | Dhp | Wb |
| CMC | Tween 20 | Sn + Dhp | Sa + Wb |
| CMC | Tween 20 | Sn | Sa + Wb |
| CMC | Tween 20 | Dhp | Sa + Wb |
| CMC | Tween 20 | Sn + Dhp | Sb |
| CMC | Tween 20 | Sn | Sb |
| CMC | Tween 20 | Dhp | Sb |
| CMC | Tween 20 | Sn + Dhp | Sb + Wb |
| CMC | Tween 20 | Sn | Sb + Wb |
| CMC | Tween 20 | Dhp | Sb + Wb |
| CMC | Tween 20 | Sn + Dhp | Talc Powder |
| CMC | Tween 20 | Sn | Talc Powder |
| CMC | Tween 20 | Dhp | Talc Powder |
| CMC | Tween 20 | Sn + Dhp | Talc Powder + Wb |
| CMC | Tween 20 | Sn | Talc Powder + Wb |
| CMC | Tween 20 | Dhp | Talc Powder + Wb |
| CMC; carboxymethyl cellulose, Sn; sodium nitrate, Dhp; dipotassium hydrogen phosphate, Sa; sodium alginate, Wb; wheat bran, Sb; sodium bentonite | | | |
