# Supplementary material for: Dual biocontrol and osmotic stress mitigation by endophytic Aspergillus micronesiensis and Penicillium momoi against fusarium pathogens
Source: PLoS One. 2026 Jul 29;21(7):e0353217. doi: 10.1371/journal.pone.0353217 (PMC13421755; doi:10.1371/journal.pone.0353217)
Supplement: S6 Table — (DOCX) [file pone.0353217.s006.docx]

| **S6 Table** | | | | | | | | | | |  |
| --- | --- | --- | --- | --- | --- | --- | --- | --- | --- | --- | --- |
| Source of variation | Degree of freedom |  | Mean squares | | | | | | |  | |
|  |  |  | Spad | Height | SFW | RFW | SDW | RDW | DS |  | |
| Block | 3 |  | 186.17^**^ | 182.01^**^ | 2.4934^**^ | 2.85^**^ | 0.062^**^ | 0.0268^**^ | 149.74^*^ |  | |
| Cultivar (A) | 1 |  | 30.71^**^ | 384.40^**^ | 0.2681^*^ | 1.28^**^ | 0.023^**^ | 0.0292^**^ | 7222.66^**^ |  | |
| Soil moisture content (B) | 1 |  | 408.64^**^ | 7290.00^**^ | 253.6381^**^ | 177.87^**^ | 3.027^**^ | 1.6618^**^ | 472.66^*^ |  | |
| Pathogen (C) | 1 |  | 1039.89^**^ | 577.60^**^ | 31.7463^**^ | 43.35^**^ | 0.684^**^ | 0.5426^**^ | 111566.41^**^ |  | |
| Treatment (D) | 4 |  | 231.71^**^ | 294.18^**^ | 11.9488^**^ | 4.15^**^ | 0.306^**^ | 0.0461^**^ | 2591.80^**^ |  | |
| A × B | 1 |  | 18.43^**^ | 3.02^ns^ | 6.9014^**^ | 1.12^**^ | 0.078^**^ | 0.0544^**^ | 191.41^ns^ |  | |
| A × C | 1 |  | 7.10^**^ | 42.02^**^ | 0.0004^ns^ | 2.08^**^ | 0.037^**^ | 0.0005^ns^ | 7222.66^**^ |  | |
| A × D | 4 |  | 17.32^**^ | 18.40^**^ | 0.9820^**^ | 0.97^**^ | 0.022^**^ | 0.0051^**^ | 142.58^ns^ |  | |
| B × C | 1 |  | 105.79^**^ | 27.22^**^ | 2.8756^**^ | 3.34^**^ | 0.060^**^ | 0.0337^**^ | 472.66^*^ |  | |
| B × D | 4 |  | 5.95^**^ | 9.53^**^ | 1.9723^**^ | 0.55^**^ | 0.035^**^ | 0.0187^**^ | 72.26^ns^ |  | |
| C × D | 4 |  | 4.54^**^ | 20.94^**^ | 2.7689^**^ | 0.74^**^ | 0.032^**^ | 0.0086^**^ | 2591.80^**^ |  | |
| A × B × C | 1 |  | 1.14^ns^ | 19.60^**^ | 2.9079^**^ | 4.42^**^ | 0.039^**^ | 0.0118^**^ | 191.41^ns^ |  | |
| A × B × D | 4 |  | 7.18^**^ | 21.65^**^ | 0.2939^**^ | 0.46^**^ | 0.013^**^ | 0.0090^**^ | 83.98^ns^ |  | |
| A × C × D | 4 |  | 2.87^*^ | 6.28^**^ | 0.4437^**^ | 0.71^**^ | 0.015^**^ | 0.0065^**^ | 142.58^ns^ |  | |
| B × C × D | 4 |  | 12.93^**^ | 0.94^ns^ | 0.7336^**^ | 0.55^**^ | 0.005^*^ | 0.0094^**^ | 72.26^ns^ |  | |
| A × B × C × D | 4 |  | 4.62^**^ | 8.98^**^ | 1.0041^**^ | 0.06^ns^ | 0.006^*^ | 0.0043^**^ | 83.98^ns^ |  | |
| Error | 117 |  | 0.88 | 0.77 | 0.0402 | 0.04 | 0.002 | 0.0003 | 58.93 |  | |
| *, ** and ns indicate significant difference p≤0.05, p≤0.01 and non-significant, respectively. | | | | | | | | | | |  |
