# Supplementary figures and images for: Dual biocontrol and osmotic stress mitigation by endophytic Aspergillus micronesiensis and Penicillium momoi against fusarium pathogens
Source: PLoS One. 2026 Jul 29;21(7):e0353217. doi: 10.1371/journal.pone.0353217 (PMC13421755; doi:10.1371/journal.pone.0353217)

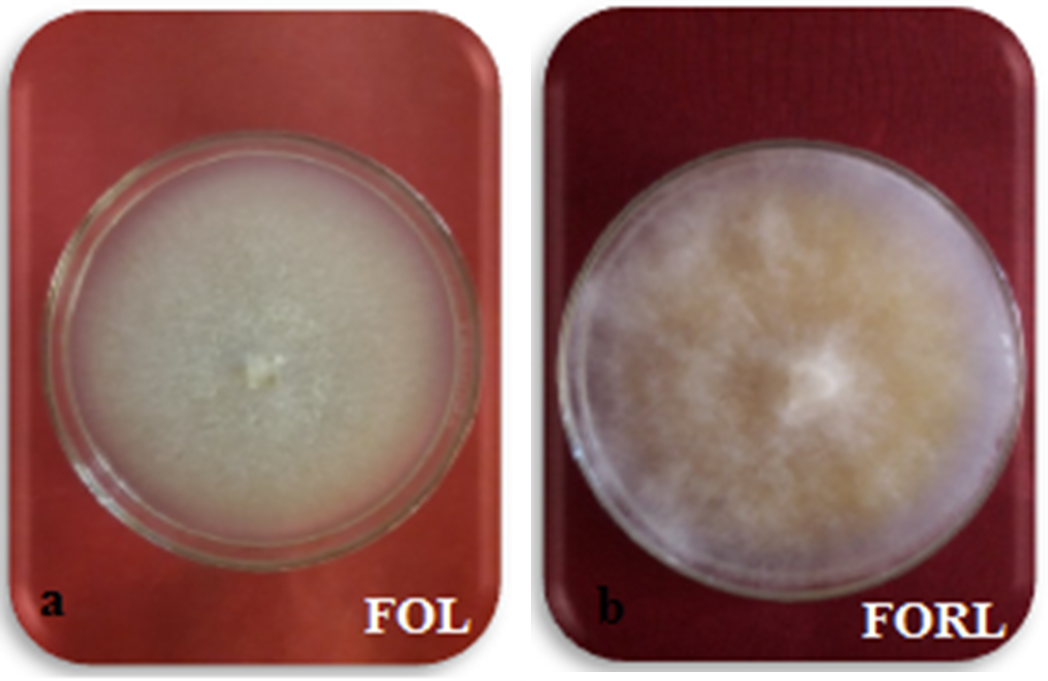

Supplement: S1 Fig — (PNG) [file pone.0353217.s007.png]

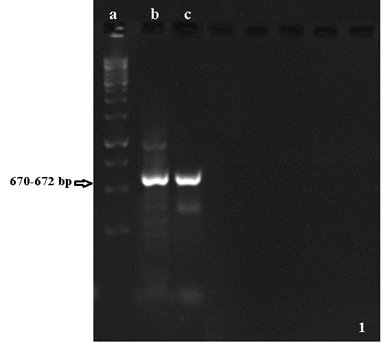

Supplement: S2 Fig — Ladder DNA 1 Kbp (a), DNA fragments (670–672 bp) of two isolates amplified with primer pair uni (b and c). (TIF) [file pone.0353217.s008.tif]

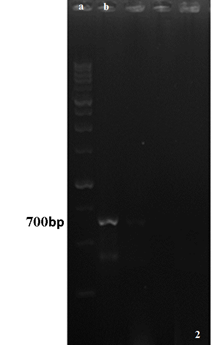

Supplement: S3 Fig — Ladder DNA 1 Kbp (a), DNA fragments (700 bp) of one isolate amplified with primer pair Spr1 (b). (PNG) [file pone.0353217.s009.png]

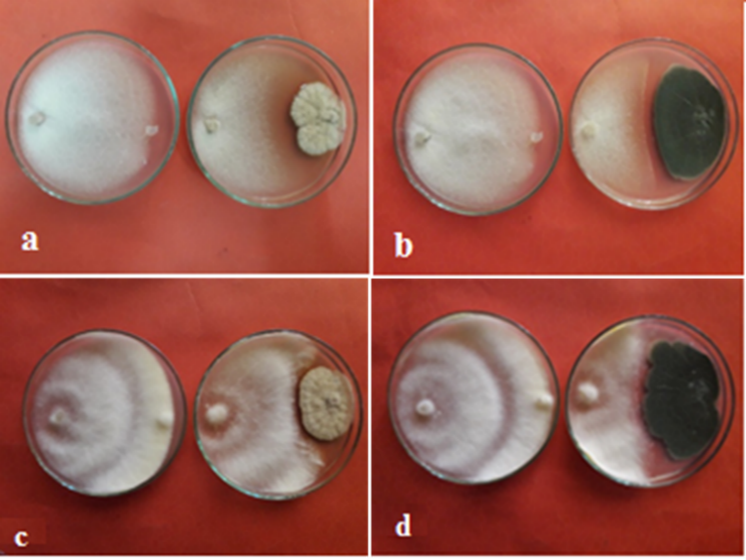

Supplement: S4 Fig — Aspergillus micronesiensis against Fusarium oxysporum f. sp. lycopersici (FOL, a). Penicillium momoi against FOL (b). A. micronesiensis against F. oxysporum f. sp. radicis-lycopersici (FORL, c). P. momoi against FORL (d). (PNG) [file pone.0353217.s010.png]

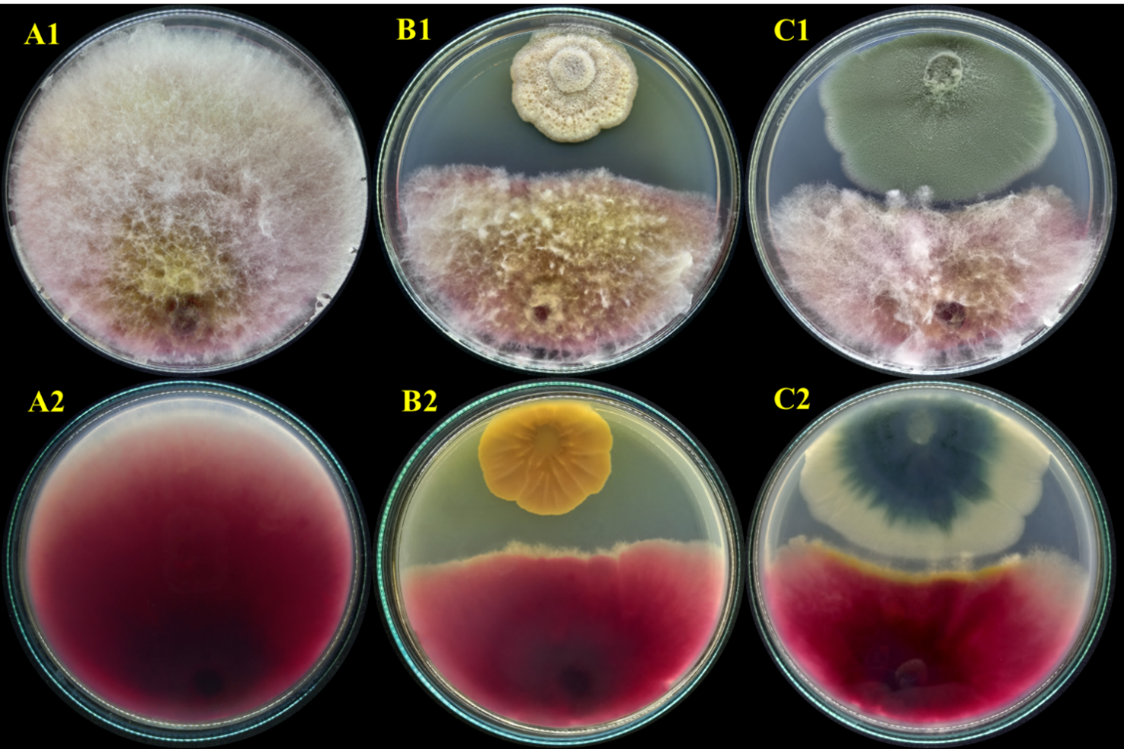

Supplement: S5 Fig — Indices 1 and 2 correspond to the top and bottom views, respectively. (PNG) [file pone.0353217.s011.png]

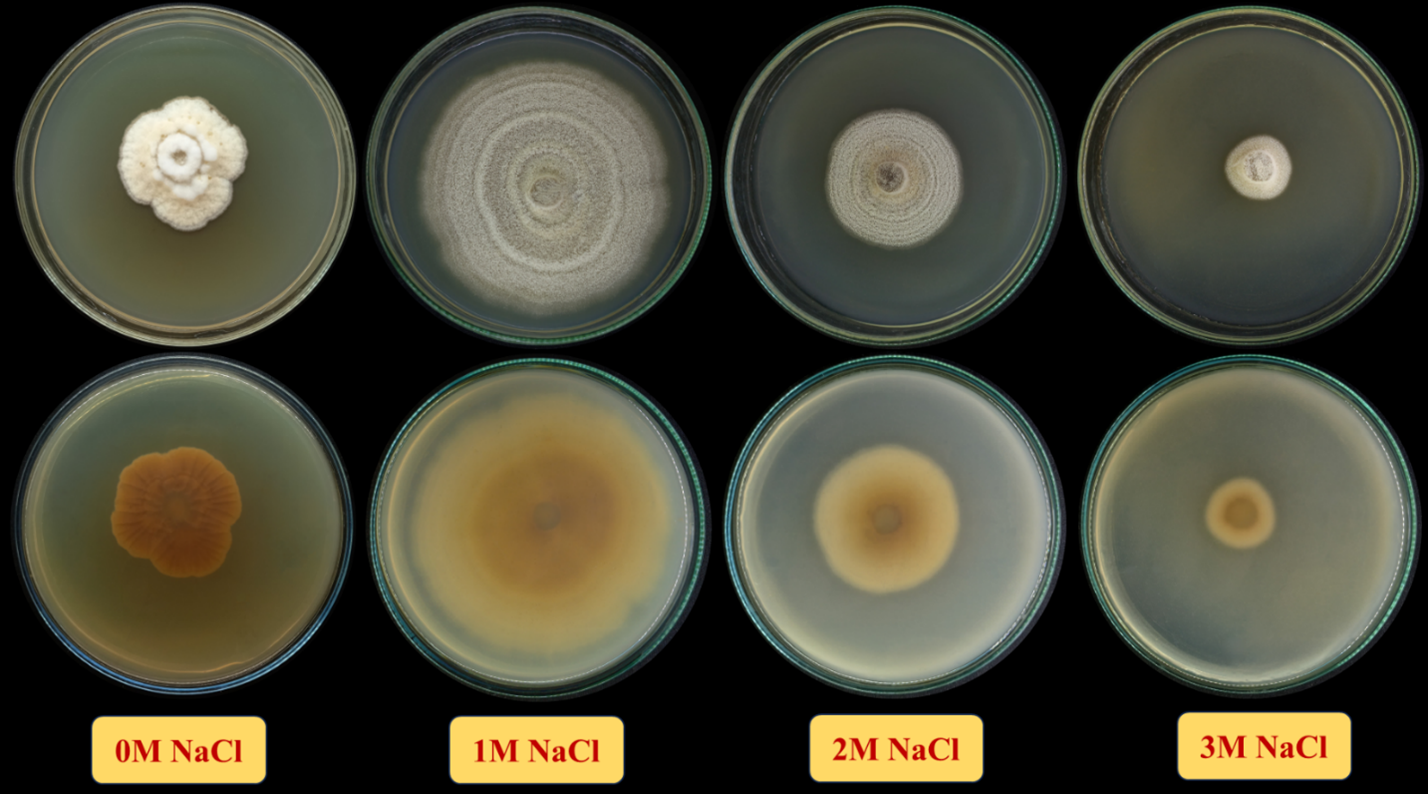

Supplement: S6 Fig — (PNG) [file pone.0353217.s012.png]

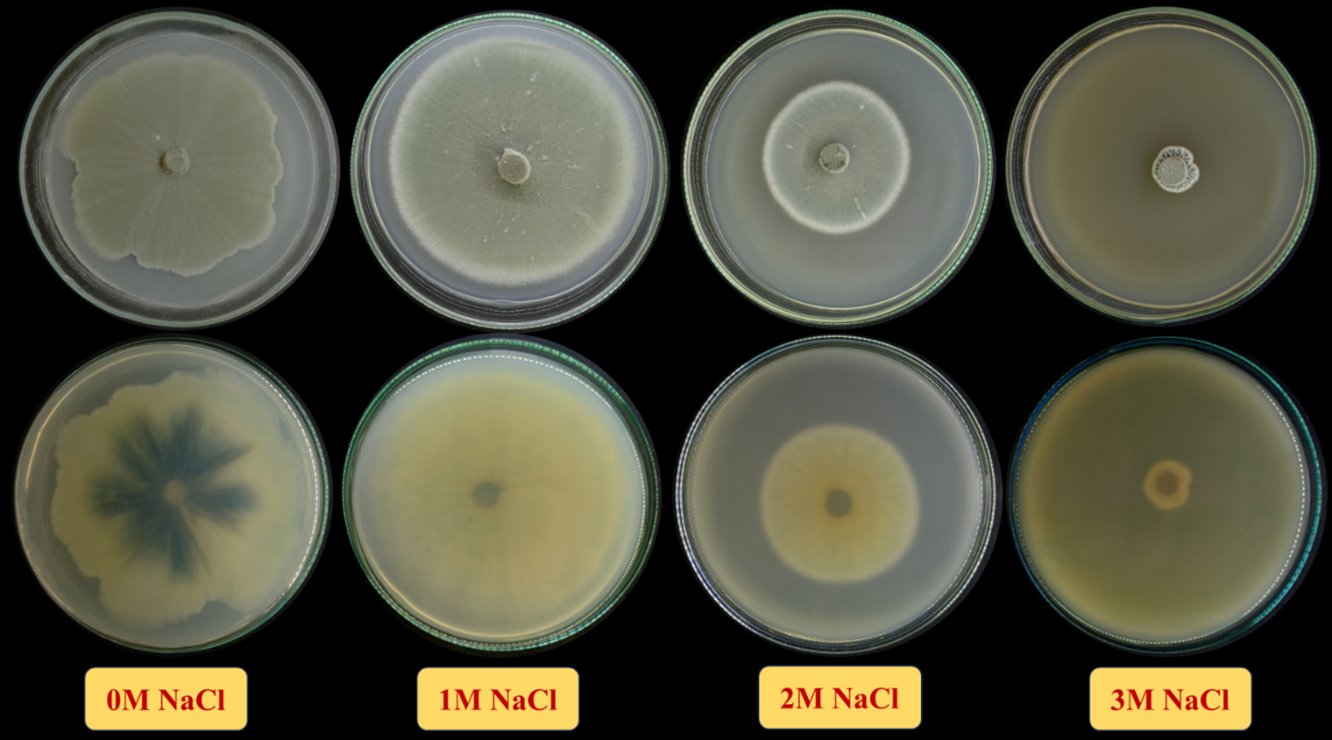

Supplement: S7 Fig — (PNG) [file pone.0353217.s013.png]

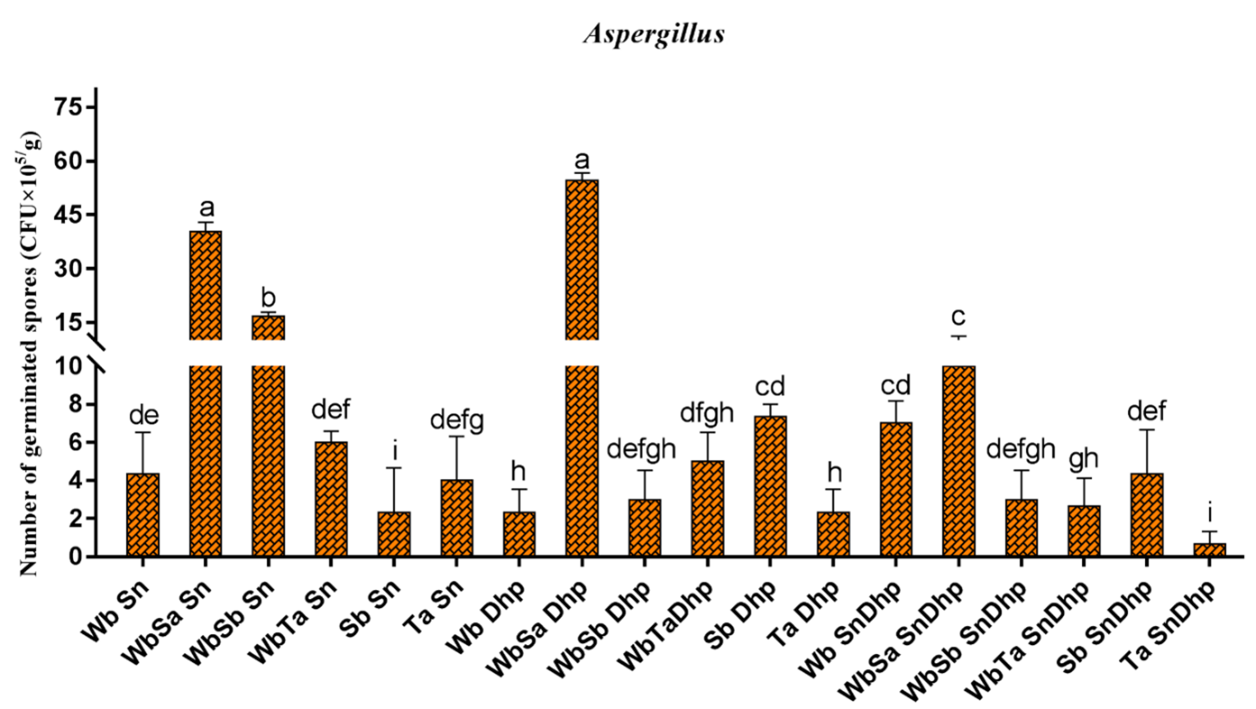

Supplement: S8 Fig — The materials used in this study included wheat bran (Wb), sodium alginate (Sa), sodium bentonite (Sb), talc (Ta), sodium nitrate (Sn), and dipotassium hydrogen phosphate (Dhp). (TIF) [file pone.0353217.s014.tif]

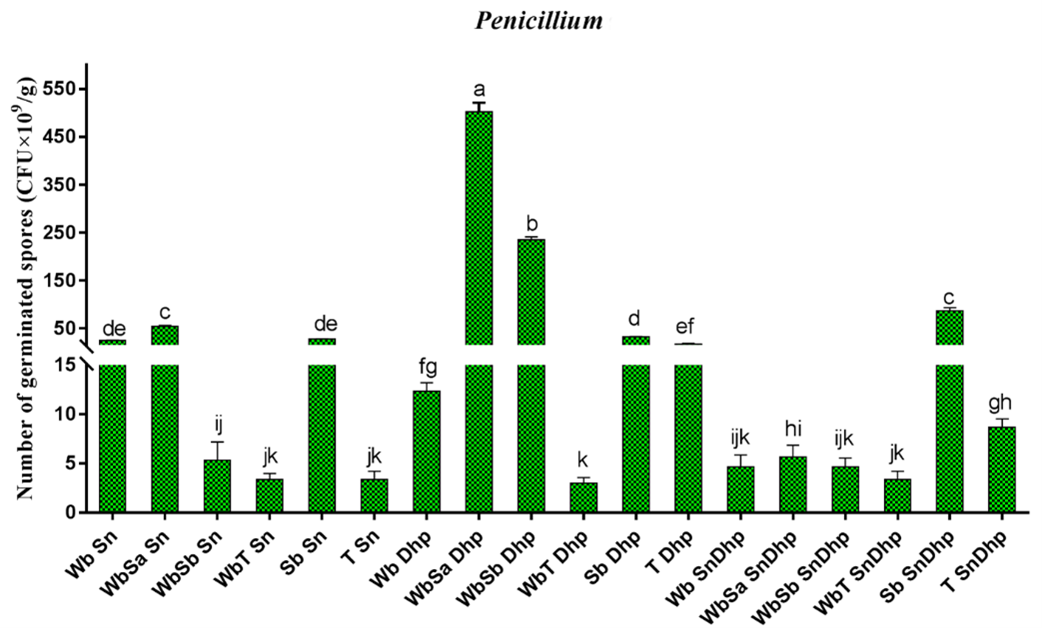

Supplement: S9 Fig — The materials used in this study included wheat bran (Wb), sodium alginate (Sa), sodium bentonite (Sb), talc (Ta), sodium nitrate (Sn), and dipotassium hydrogen phosphate (Dhp). (TIF) [file pone.0353217.s015.tif]

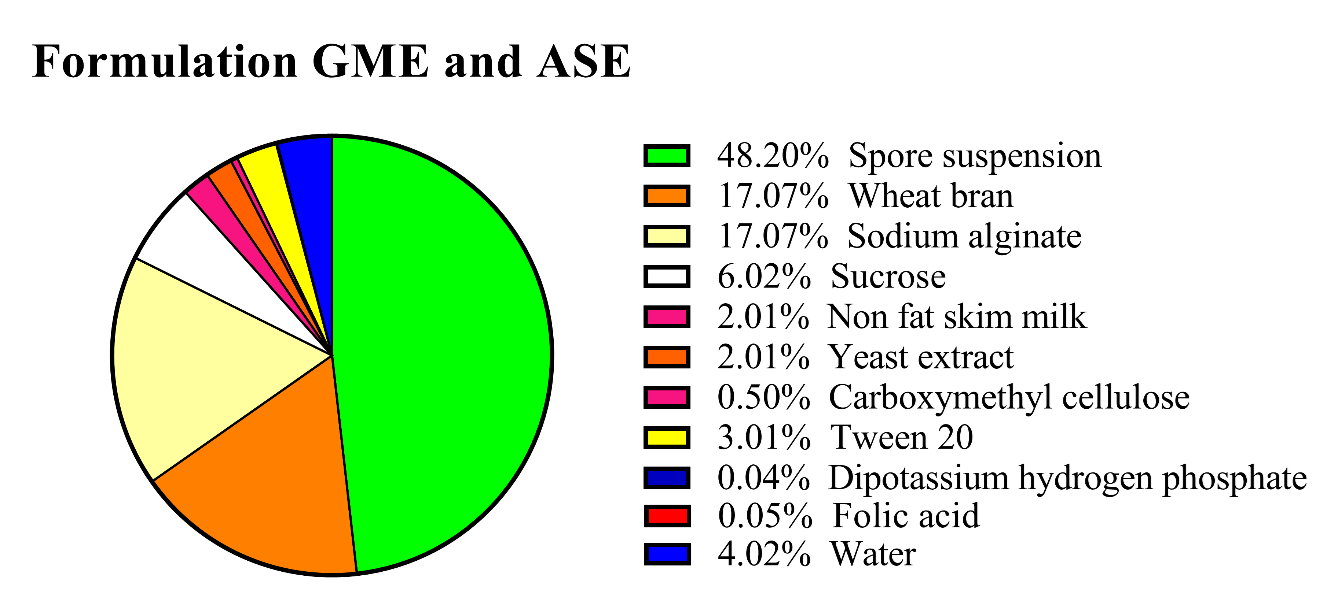

Supplement: S10 Fig — (TIF) [file pone.0353217.s016.tif]
